# Effects of mind-body training on upper-limb function in stroke patients: a multilevel dose-response meta-analysis

**DOI:** 10.3389/fmed.2026.1827942

**Published:** 2026-06-12

**Authors:** Lin Luo, Hengjun Zhu, Chengyu Zhou, Wang Yu, Zheng Wang

**Affiliations:** 1 Department of Physical Education, North China Electric Power University, Beijing, China; 2 Central Military Commission Training Management Department Training Management Center, Beijing, China; 3 Strength and Conditioning Training College, Beijing Sports University, Beijing, China; 4 Capital University of Physical Education and Sports, Beijing, China; 5 Department of Physical Education, Hebei University, Hebei, China

**Keywords:** dose–response relationship, meta-analysis, mind-body training, stroke, upper-limb function

## Abstract

**Objective:**

This systematic review and meta-analysis examined the effects of mind-body training (MBT) on upper-limb function in patients with stroke. A Bayesian dose-response model and an XGBoost-SHAP analysis were used to assess the optimal intervention dosage and to determine key factors influencing outcomes.

**Methods:**

PubMed, Web of Science, PsycINFO, and the Cochrane Library were searched from their inception to January 10, 2026, for randomized controlled trials. Effect sizes were calculated as standardized mean differences (SMDs). Risk of bias was assessed using ROB2. Analyses included Egger's test, trim-and-fill, sensitivity analysis, subgroup analysis, meta-regression, trial sequential analysis, and Bayesian hierarchical dose-response modeling. XGBoost-SHAP was used for exploratory analysis of predictors.

**Results:**

*Eighteen* RCTs were included, of which 16 contributed to the meta-analysis (769 participants). After removing influential studies, MBT significantly improved upper-limb function (SMD = 0.65, 95% CI 0.40–0.91, *P* < 0.001). Heterogeneity was high (*I*^2^ = 87.3%), but Egger's test indicated no publication bias (*P* = 0.753). GRADE assessment showed moderate-certainty evidence. Exploratory subgroup analyses suggested larger effects in studies involving subacute patients (SMD = 0.95) and Tai Chi interventions (SMD = 0.99). Nonlinear dose-response analysis revealed an inverted U-shaped relationship, with an optimal cumulative duration of about 33.5 h (range: 32–35 h), corresponding to 5 sessions per week × 30 min × 14–15 weeks. Exploratory XGBoost-SHAP analysis indicated that patient type had the highest relative importance among the study-level predictors included.

**Conclusion:**

Moderate-certainty evidence indicates that MBT effectively enhances upper-limb function in stroke patients, with the best results seen at around 33.5 total h. A schedule of 30-min sessions, five times a week for 14–15 weeks, may provide preliminary guidance for future dose-stratified trials and clinical planning, particularly in studies involving subacute-stage patients, although this finding should be confirmed in future stage-stratified RCTs.

**Systematic review registration:**

https://www.crd.york.ac.uk/PROSPERO/view/CRD420261334249, identifier: CRD420261334249.

## Introduction

Stroke is a major cerebrovascular disorder and remains one of the leading causes of death and long-term disability worldwide. According to the 2021 Global Burden of Disease Study, there were 93.8 million prevalent strokes and 11.9 million incident strokes worldwide. These data underscore the substantial and ongoing burden on acute care, rehabilitation services, long-term nursing care, and family caregiving (1). Pathophysiologically, ischemic stroke primarily results from interruption of cerebral blood flow due to thrombosis or embolism. This reduction in oxygen and glucose supply leads to ATP depletion, energy failure, ionic imbalance, excitotoxicity, mitochondrial dysfunction, oxidative stress, neuroinflammation, blood-brain barrier impairment, and ultimately, neural cell death (2). These pathological processes disrupt the corticospinal and sensorimotor networks that support voluntary movement, thereby contributing to motor dysfunction after a stroke. Upper-limb dysfunction is especially prevalent and difficult to recover from after such an event. In addition to these classical injury cascades, recent evidence suggests that progranulin (PGRN), a multifunctional protein involved in inflammation, wound healing, and nervous system development, may play a significant role in the pathogenesis of ischemic stroke. Mature PGRN appears to exert anti-inflammatory and neuroprotective effects, whereas its derivative, granulin, may promote pro-inflammatory cytokine expression. Consequently, PGRN-related pathways may be implicated in post-ischemic neuroinflammation, neural repair, and long-term functional recovery (3). Post-stroke upper-limb dysfunction commonly presents as decreased muscle strength, impaired fine motor skills, and increased spasticity, all of which directly affect patients' ability to perform daily activities such as eating, dressing, and maintaining personal hygiene (4, 5). Therefore, upper-limb functional recovery is considered a crucial indicator for evaluating stroke rehabilitation outcomes and long-term prognosis (5).

Epidemiological studies show that roughly one-third of stroke patients still experience lasting functional impairments after the acute phase, requiring ongoing dependence on rehabilitation interventions (6). Patients with functional impairments had a significantly higher risk of death, with mortality increasing from 6.9 to 19.8% (7). Furthermore, post-stroke functional impairment is closely associated with the risk of recurrence (8). These findings demonstrate that effective interventions targeting functional impairment in stroke patients are crucial not only for individual recovery but also for public health concerns.

Exercise training is a vital non-drug treatment used in stroke patient rehabilitation. Systematic reviews have demonstrated that structured exercise can significantly improve motor skills and overall functional ability in individuals who have experienced a stroke (9, 10). Traditional rehabilitation training has often emphasized repetitive limb movement practice but has not adequately addressed attentional engagement and psychological regulation. This oversight has led to limited patient adherence and a lack of long-term commitment to rehab efforts (11). Against this background, MBT, which emphasizes the integration of mental engagement, breath regulation, and coordinated body movements, has gradually gained more attention. MBT is a type of exercise focused on coordinating the mind and body, with representative modalities such as Baduanjin and Tai Chi (12, 13). These training modalities involved smooth, rhythmic movements and moderate intensity, making them appropriate for long-term use in stroke patients during periods of functional limitation (14). Research in neurorehabilitation shows that MBT can enhance central nervous system plasticity through bilateral symmetrical movements, sensory feedback, and attentional regulation, leading to improvements in both motor and cognitive functions (15, 16).

Existing RCTs and systematic reviews show that MBT can enhance balance and lower-limb sensorimotor function in stroke patients (17), and may also enhance cognitive function (18). However, previous studies have primarily focused on overall upper- and lower-limb motor function or balance as primary outcome measures, highlighting a clear gap in the independent analysis of upper-limb function (17). At the same time, considerable differences were observed across studies in intervention types, training frequencies, and durations, resulting in limited consistency in the results. Additionally, standardized scales for assessing upper-limb function, such as the Fugl–Meyer Assessment (FMA) for the upper extremity, still require systematic review and quantitative analysis (19, 20).

Considering the important role of upper-limb function in stroke patients and the gaps in current evidence regarding outcomes and systematic integration, this study synthesized findings from RCTs through a systematic review and meta-analysis. The goal was to clarify how MBT affects upper-limb function in stroke patients, providing solid, evidence-based support for its use in stroke rehabilitation. Additionally, this study aims to help develop safe, practical, and scalable rehabilitation methods in clinical practice.

## Materials and methods

### Study design

This article offers a systematic review of RCTs and adheres to the Preferred Reporting Items for Systematic Reviews and Meta-Analyses (PRISMA) guidelines (21). Before beginning the screening of search results, the protocol was registered with the International Prospective Register of Systematic Reviews (PROSPERO) under registration number CRD420261334249.

### Study Inclusion Criteria

The inclusion criteria for this study were as follows: (1) only RCTs assessing the effects of MBT on upper-limb function in stroke patients were included; (2) the intervention group participated in a structured MBT program, while the control group received either no intervention or standard therapy; (3) the study population was limited to stroke patients without restrictions based on sex, age, race, or socioeconomic status; (4) the primary outcome measures included validated scales evaluating upper-limb or motor function, such as the FMA, the Barthel Index of Activities of Daily Living (BI), the Arm Curl Test (ACT), and the 9-Hole Peg Test (9HPT). To ensure data comparability, data were collected at the immediate post-intervention time point, and (5) studies had to be published in English or Chinese and available in full text. The exclusion criteria included non-experimental studies, non-clinical studies, secondary literature (such as systematic reviews and meta-analyses), non-original works, and gray literature that is not peer-reviewed. Although systematic reviews did not meet the inclusion criteria, their reference lists were checked for potential eligible studies.

### Search strategy

This study systematically searched the PubMed, Web of Science, PsycINFO, and Cochrane Library databases up to January 10, 2026, to comprehensively identify RCTs concerning MBT for improving upper-limb function in stroke patients. The search strategy was developed using the PICOS framework, focusing on stroke patients as the population (P), MBT (defined as at least one session) as the intervention (I), a blank control or usual training as the comparator (C), changes in standardized upper-limb function scale scores as the primary outcome (O), and RCTs as the study design (S). Keywords and subject headings were combined, for example: [“Stroke”(Mesh) OR stroke(Title/Abstract) OR “cerebrovascular accident”(Title/Abstract)] AND [“Mind-Body Therapies”(Mesh) OR “mind-body”(Title/Abstract) OR “physical and mental training”(Title/Abstract) OR “Baduanjin”(Title/Abstract) OR “Tai Chi”(Title/Abstract) OR “Qigong”(Title/Abstract) OR “Yoga”(Title/Abstract) OR meditation(Title/Abstract)] AND [“Upper Extremity”(Mesh) OR “upper limb”(Title/Abstract) OR “upper extremity”(Title/Abstract) OR “arm function”(Title/Abstract)] AND [randomized controlled trial(Publication Type) OR randomized(Title/Abstract) OR randomly(Title/Abstract)].

### Selection process

This study strictly adhered to the PRISMA guidelines during the literature screening process. All retrieved records were imported into Zotero 7.0 to automatically remove duplicates. A two-stage independent screening process was then performed. In the initial stage, two reviewers independently and blindly screened the titles and abstracts to exclude studies that clearly did not meet the inclusion criteria. During the full-text screening stage, the complete texts of studies that passed the initial screening were downloaded and further assessed for eligibility using the PICOS framework. Any disagreements during the review process were resolved through consultation with a third scholar, thereby reducing potential selection bias. A standardized form was used to extract data from the included studies, and two researchers independently entered the data. Key variables, such as sample characteristics and intervention details, were cross-verified. Furthermore, detailed components of the intervention from each included study were extracted, and all relevant information was summarized in a supplementary file. When the original study did not provide complete information on the specific exercise procedures, the procedures were uniformly marked as ‘not reported' without subjective inference. All disagreements were resolved through discussion and consensus among a panel of three researchers. Detailed movement components and the completeness of reporting for the included MBT programs are summarized in the Supplementary Document.

### Data synthesis

All statistical analyses were performed using R version 4.3.3, primarily utilizing the meta, metafor, and ggplot2 packages. For continuous outcome measures, the SMD was chosen as the effect size, given the study's scales, with Hedges' g correction applied. Effect sizes were classified as large when g ≥ 0.8, moderate when *g* = 0.5, and small when *g* = 0.2 (22). Effect sizes were combined using inverse-variance weighting, with the restricted maximum likelihood (REML) random-effects model selected as the primary analytical approach. When heterogeneity was low (*I*^2^ < 50%), results from fixed-effect models were also presented. Heterogeneity was evaluated with the Q test (*P* < 0.10 indicating significance) and the *I*^2^ statistic (>50% indicating substantial heterogeneity) (23). Publication bias was assessed using funnel plots and Egger's regression test, and the trim-and-fill method was used to address data asymmetry. Outlier studies were identified using standardized residuals (|Z| > 2.5) and Cook's distance (>3 times the mean) (24). The sensitivity analysis strategy included three main parts: (1) sequentially removing studies using the metainf function; (2) performing REML-based meta-regression analyses to examine the relationships between moderator variables—such as intervention duration and population type—and effect sizes, with bubble plots used for visualization (25); and (3) performing subgroup analyses to identify potential sources of heterogeneity. For subgroup analyses by disease stage, stroke phases were classified by time elapsed since stroke onset, when available. In accordance with the recommendations of the Stroke Recovery and Rehabilitation Roundtable, the subacute phase was defined as the period from the 1st week after stroke onset to within 6 months of stroke onset, and the chronic phase was defined as occurring more than 6 months after stroke onset (26). When the original study reported only the disease stage without specifying an exact onset time, we preserved the authors' classification and cross-verified it against the available participant descriptions. Consequently, the subgroup analysis by disease stage should be interpreted as an exploratory study-level analysis rather than an individual-patient-level analysis. To standardize data across various scales, the Hedges–Olkin formula was used to transform the raw data: $SMD=\frac{M_{Intervention}-M_{Control}}{SD_{Pooled}},\;SD_{Pooled}=\sqrt{\frac{{\left(n_{1}-1\right)SD}_{1}^{2}+{\left(n_{2}-1\right)SD}_{2}^{2}}{n_{1}+n_{2}-2}}\;$(27). To improve estimate accuracy in areas with small sample sizes or limited exposure data, we used a Bayesian hierarchical dose-response model with restricted cubic splines. This method incorporated weakly informative priors and applied Markov Chain Monte Carlo (MCMC) estimation techniques (28, 29). Because some studies may have included multiple correlated effect sizes from the same source, treating them as completely independent observations could underestimate the standard errors and decrease the accuracy of the pooled estimates. To further verify the robustness of the main findings, this study added a multilevel meta-analytic model to the usual random-effects meta-analysis, grouping effect sizes within each study to address within-study dependence. The pooled effect size, 95% confidence interval, and heterogeneity indices were reported as an additional comparison alongside the results of the conventional model (30). Additionally, an exploratory XGBoost ensemble learning method was used to build a dose–effect prediction model. The input features were one-hot encoded for categorical variables and standardized for continuous variables. Hyperparameters, specifically max_depth set to 3 and eta set to 0.05, were fine-tuned using three-fold cross-validation to improve the model's performance. SHAP values were used to measure each variable's marginal impact on the SMD; continuous variables were ranked by their mean absolute SHAP values. In contrast, categorical variables were evaluated by summing the SHAP values of their dummy variables. The importance of features and nonlinear relationships was shown with bar plots and swarm plots, respectively (31, 32).

### Risk of bias (quality) assessment

The revised Cochrane Risk of Bias tool for Randomized Trials (ROB2, 2019) was used to assess the risk of bias in the included RCTs. This evaluation covered five key areas: (1) the randomization process; (2) deviations from the intended interventions; (3) missing outcome data; (4) measurement of outcomes; and (5) selection of reported results (33). Two researchers independently evaluated the risk of bias using a three-level classification system: “Low Risk”, “Some Concerns”, and “High Risk”. Disagreements in evaluations were resolved through group discussion. If they couldn't reach an agreement, the disputed study was referred to a third researcher for arbitration.

## Results

### Study selection

**Figure 1:**
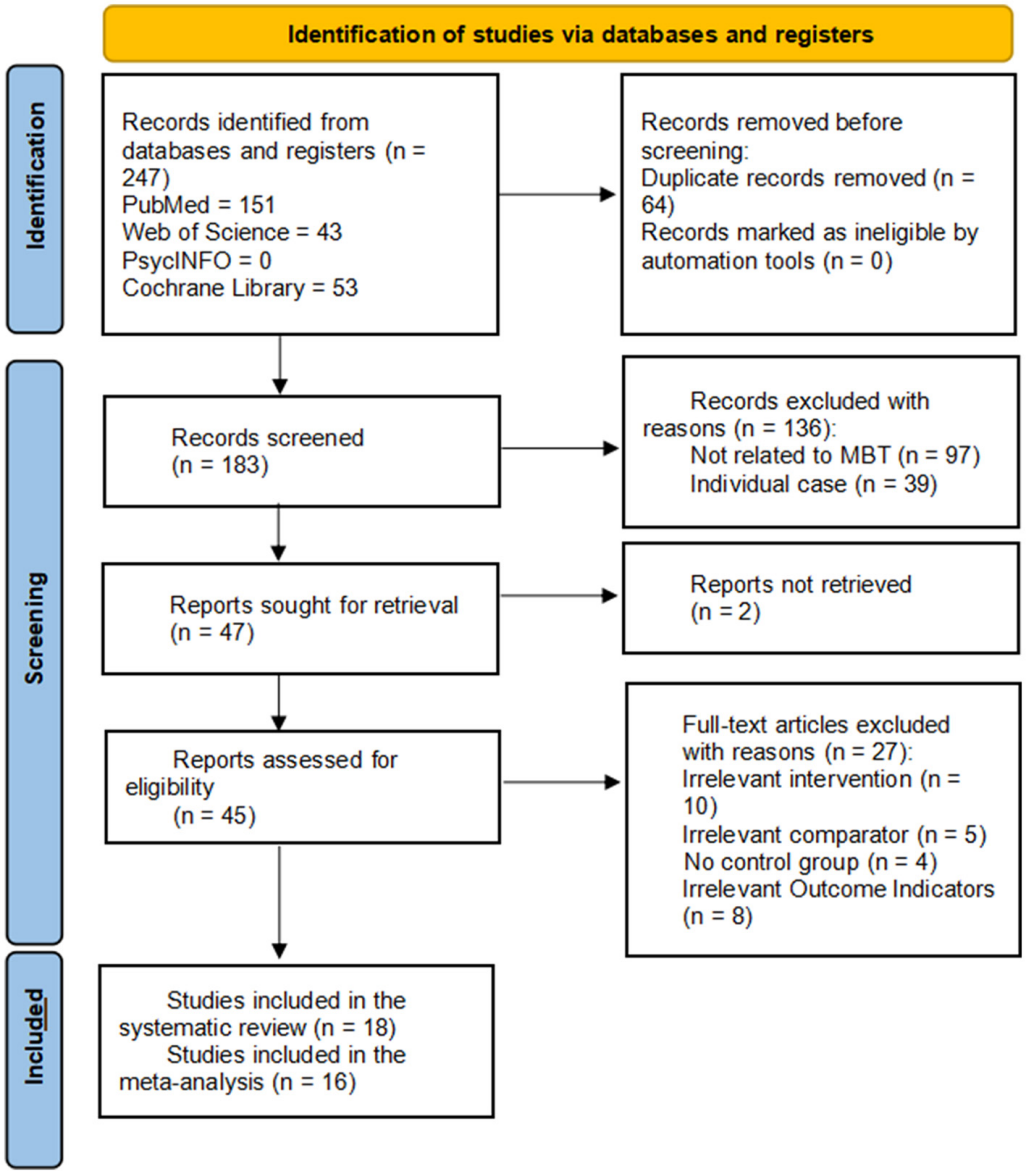
Flow diagram of the selection process. Two of the 18 studies in the systematic review were excluded from the primary pooled analysis after influence diagnostics (standardized residuals and Cook's distance), and were retained in the systematic review.

This study initially identified 247 relevant records through searches across four major databases. After removing 64 duplicates, 183 records remained for screening based on title and abstract. At this stage, 136 records were excluded for failing to meet the inclusion criteria, leaving 47 reports for full-text retrieval. Of these, 45 articles were ultimately assessed for full-text eligibility, except for 2 reports whose full texts could not be obtained. Based on the exclusion criteria shown in Figure 1, an additional 27 articles were excluded, resulting in 18 RCTs included in the systematic review, of which 16 were part of the meta-analysis (34–51).

### Risk of bias of included studies

**Figure 2:**
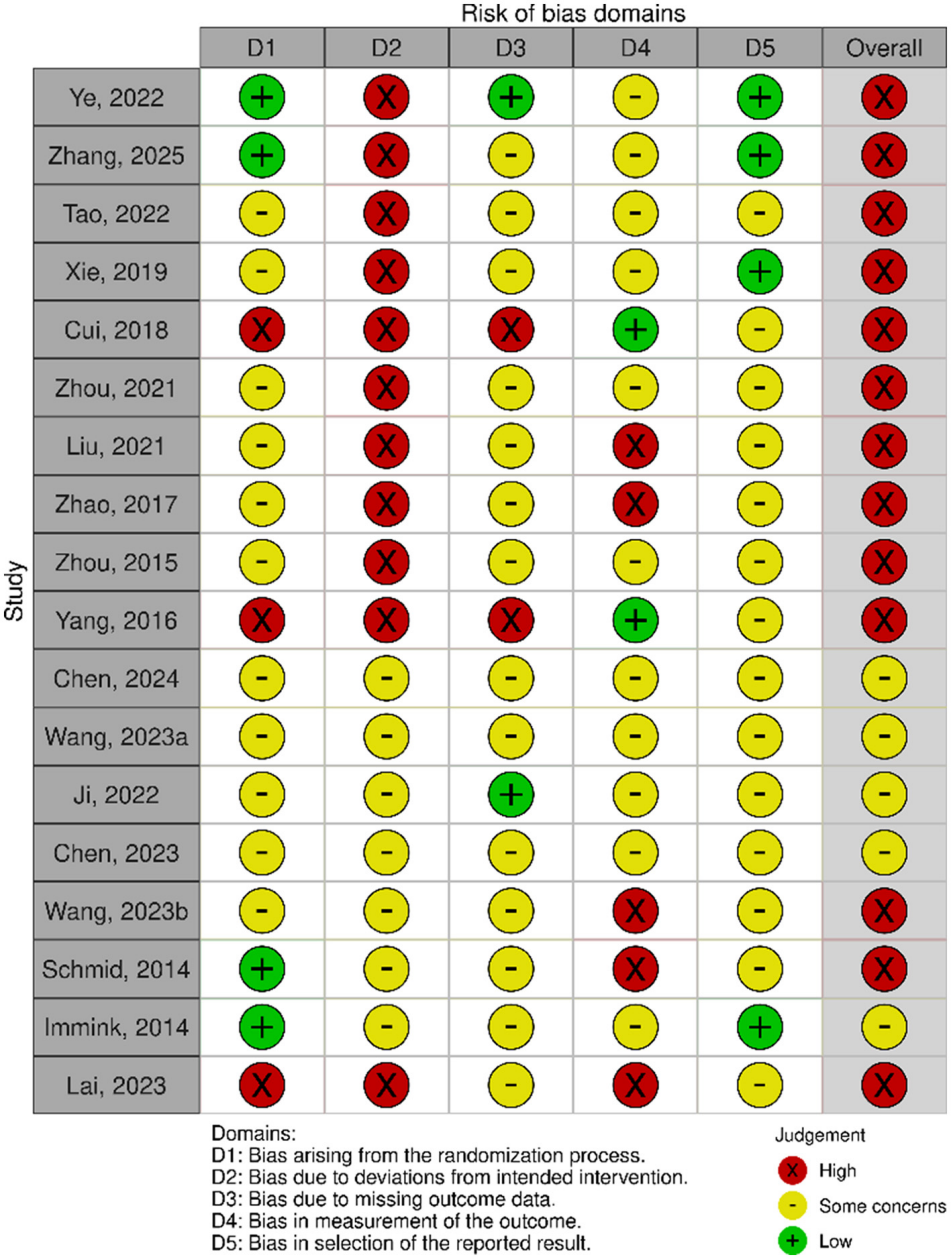
Risk of bias graph.

**Figure 3:**
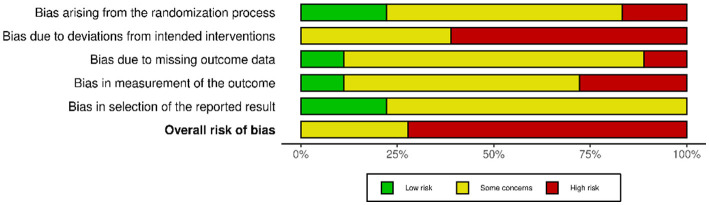
Risk of bias summary.

The risk-of-bias assessment results for the included studies are shown in Figures 2, 3, where a red “X” indicates high risk, a yellow “–” indicates some concerns, and a green “+” indicates low risk. Two reviewers independently assessed the 18 RCTs across the five domains of RoB 2.0: D1 (randomization process), D2 (deviations from intended interventions), D3 (missing outcome data), D4 (measurement of the outcome), and D5 (selection of the reported result). Disagreements were resolved through discussion and, when needed, settled by a third reviewer. The inter-rater agreement across domains was as follows: For D1 (randomization process), the simple agreement rate was 94.4% (17/18), Cohen's κ = 0.903, and weighted κ = 0.916, indicating almost perfect agreement. For D2 (deviations from intended interventions), the simple agreement rate was 100.0% (18/18), Cohen's κ = 1.000, and weighted κ = 1.000, indicating perfect agreement. For D3 (missing outcome data), the simple agreement rate was 94.4% (17/18), Cohen's κ = 0.864, and weighted κ = 0.873, indicating nearly perfect agreement. For D4 (measurement of the outcome), the simple agreement rate was 94.4% (17/18), Cohen's κ = 0.898, and weighted κ = 0.820, indicating nearly perfect agreement. For D5 (selection of the reported result), the simple agreement rate was 100.0% (18/18), Cohen's κ = 1.000, and weighted κ = 1.000, indicating perfect agreement.

Overall, inter-rater agreement was high, with all κ values≥ 0.86, indicating strong reliability of the double-masked independent assessments. Regarding bias distribution, most studies were rated as low risk in the domain of selective reporting (D5), suggesting that reporting transparency was generally adequate. However, in the areas of the randomization process (D1) and measurement of the outcome (D4), several studies were rated as having “some concerns” or “high risk” due to incomplete reporting of random sequence generation or allocation concealment procedures, as well as limited information on assessor blinding. In the area of missing outcome data (D3), some studies experienced loss to follow-up or did not clearly explain how missing data were handled, resulting in variation in follow-up completeness.

Based on these bias patterns, we cautiously interpreted the main analysis and subgroup and dose-response analyses, while considering potential systematic errors from D1 and D4. In sensitivity analyses and certainty-of-evidence assessments (e.g., GRADE), these potential sources of bias were considered critical to ensuring the robustness of the results and conclusions.

### Study characteristics

This study included 18 RCTs (34–51), with their detailed characteristics summarized in Table 1. Geographically, most studies were conducted in China, with data from the United States and Australia. Most participants were between 51 and 69 years old, spanning both subacute and chronic stroke stages. The interventions mainly involved MBT therapies like Tai Chi, yoga, and Baduanjin. At the same time, control groups generally received treatment as usual (TAU), medication alone, other active therapies, or were on a waiting list. The intervention frequency was typically 5 to 6 sessions per week, lasting from 4 to 24 weeks. The outcome measures were quite diverse, mainly comprising the FMA, hand grip strength (HGS), BIA, ACT, and 9HPT.

**Table 1:** Characteristics of the studies in the systematic review and meta-analysis.

Author/Year	Country	Design	Sample (T/C)	Mean age	Subject type	Intervention (T/C)	Protocol	Tools
Ye et al. (34)	China	RCT	24/24	62.19	Chronic stroke	Baduanjin vs. Active Control	40 min/session, 3 × /week, 24 weeks	FMA
Zhang et al. (35)	China	RCT	29/48	61.27	Subacute stroke	Tai Chi vs. Usual Care Control	60 min/session, 5 × /week, 12 weeks	FMA
Tao et al. (36)	China	RCT	40/38	62.63	Chronic stroke	Baduanjin vs. Usual Care Control	20 min/session, 5 × /week, 2 weeks	FMA
Lai et al. (37)	China	RCT	16/16	59.02	Chronic stroke	Yoga vs. Usual Care Control	60 min/session, 2 × /week, 8 weeks	HGS
Yang and Tang (38)	China	RCT	28/21	53.14	Subacute stroke	Tai Chi vs. Usual Care Control	40 min/session, 5 × /week, 8 weeks	FMA
Zhao et al. (39)	China	RCT	30/30	52.62	Subacute stroke	Tai Chi vs. Usual Care Control	30 min/session, 5 × /week, 8 weeks	FMA
Zhou et al. (40)	China	RCT	11/11	52.5	Subacute stroke	Tai Chi vs. Usual Care Control	60 min/session, 5 × /week, 4 weeks	FMA
Cui et al. (41)	China	RCT	24/21	53.4	Subacute stroke	Baduanjin vs. Usual Care Control	45 min/session, 5 × /week, 8 weeks	FMA
Xie et al. (42)	China	RCT	20/20	52.53	Subacute stroke	Baduanjin vs. Usual Care Control	50 min/session, 5 × /week, 3 weeks	FMA
Liu et al. (43)	China	RCT	30/30	57.22	Subacute stroke	Baduanjin vs. Usual Care Control	45 min/session, 3 × /week, 4 weeks	FMA
Zhou et al. (44)	China	RCT	35/35	69.3	Subacute stroke	Baduanjin vs. Usual Care Control	60 min/session, 5 × /week, 12 weeks	FMA
Ji et al. (45)	China	RCT	45/45	68.87	Subacute stroke	Baduanjin vs. Usual Care Control	30 min/session, 6 × /week, 8 weeks	FMA
Chen and Liu (46)	China	RCT	40/40	62.37	Chronic stroke	Baduanjin vs. Active Control	40 min/session, 4 × /week, 12 weeks	FMA
Wang et al. (47)	China	RCT	9/8	51	Subacute stroke	Tai Chi vs. Active Control	30 min/session, 7 × /week, 4 weeks	FMA
Wang et al. (48)	China	RCT	30/30	63.95	Subacute stroke	Baduanjin vs. Active Control	20 min/session, 6 × /week, 4 weeks	BIA
Chen et al. (49)	China	RCT	21/21	53.5	Chronic stroke	Baduanjin vs. Usual Care Control	45 min/session, 5 × /week, 4 weeks	FMA
Schmid et al. (50)	USA	RCT	37/10	63.1	Chronic stroke	Yoga vs. Usual Care Control	60 min/session, 2 × /week, 8 weeks	ACT
Immink et al. (51)	Australia	RCT	11/11	59.6	Chronic stroke	Yoga vs. Usual Care Control	47 min/session, 7 × /week, 10 weeks	9HPT

RCT, randomized controlled trial; T, intervention group; C, control group; Chronic stroke refers to patients who have experienced a stroke more than 6 months prior, while subacute stroke pertains to patients within the time frame of 7 days to 6 months post-stroke onset. If the exact onset time was not reported, the classification provided by the original study was retained. Usual Care Control: Standard treatment; Active Control: Other active treatment approaches besides standard treatment; FMA, Fugl-Meyer assessment score; FMA (Total): Fugl-Meyer Assessment total score; HGS, hand grip strength; BIA, Barthel index of ADL; ACT, Arm curl test; 9HPT, the 9-Hole Peg Test; Studies by Wang et al. (47) and Wang et al. (48) published in the same year by different authors.

### Meta-analysis

**Figure 4:**
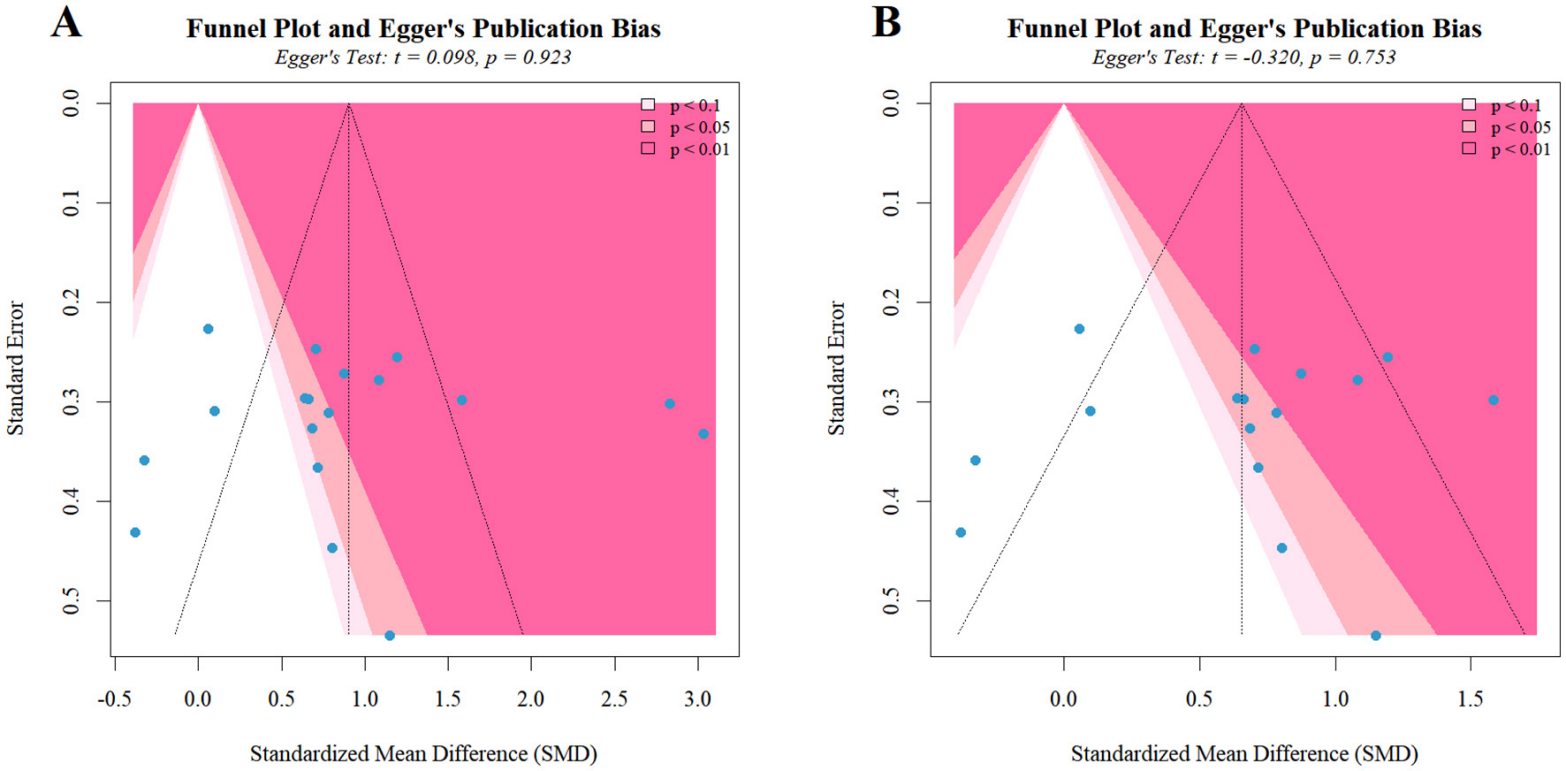
Funnel plot and Egger's test for publication bias. **(A)** shows the results before outlier removal, and **(B)** shows the results after outlier removal.

**Figure 5:**
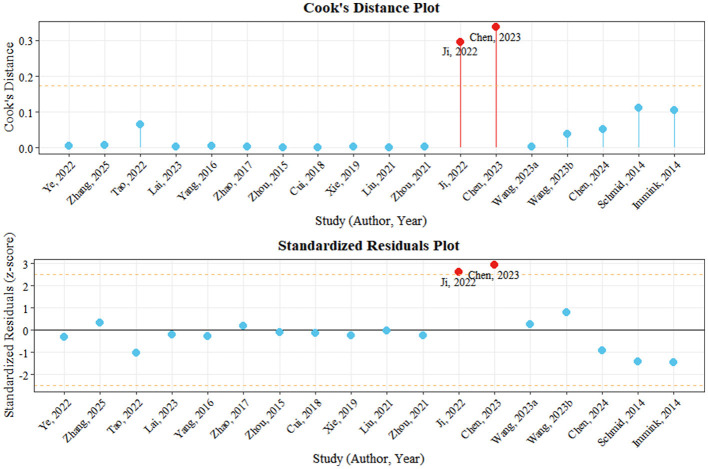
Influence diagnostics of the included studies using standardized residuals and Cook's distance. Studies by Wang et al. (47) and Wang et al. (48) were published in the same year by different authors

**Figure 6:**
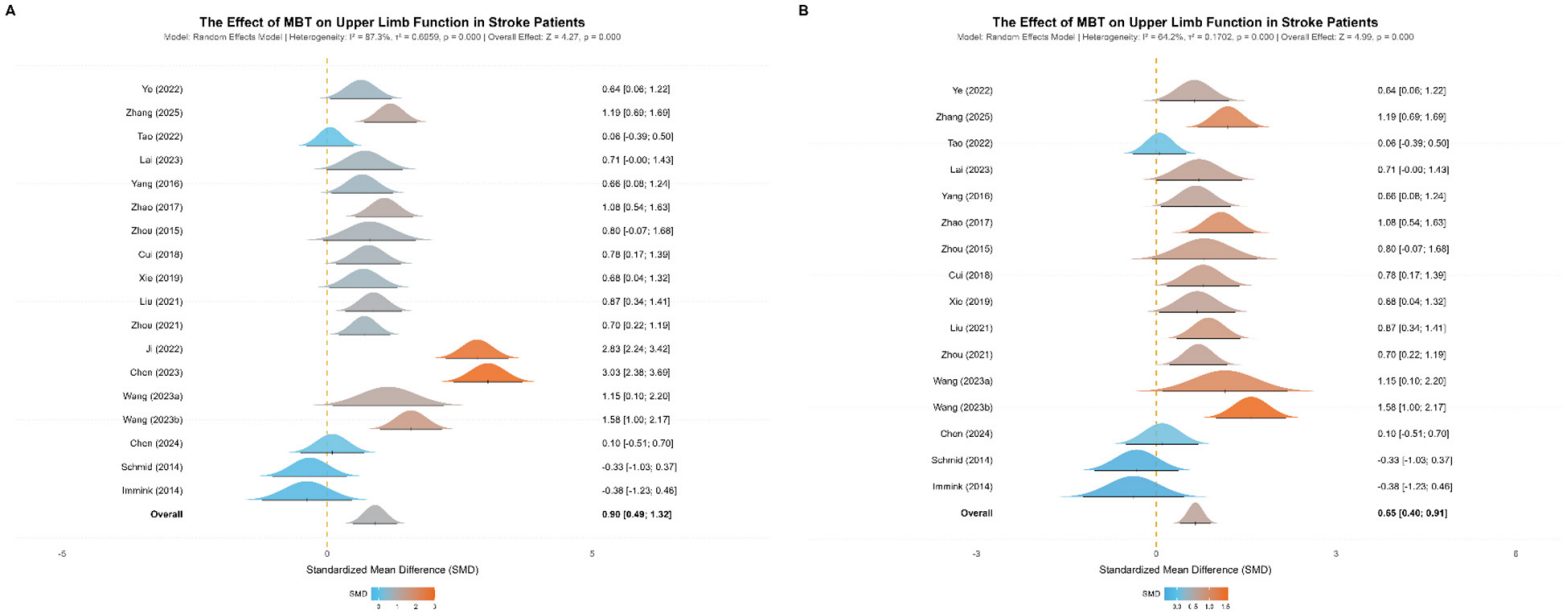
Forest plot of the effects of mind-body training on upper-limb function in patients with stroke. Studies by Wang et al. (47) and Wang et al. (48) were published in the same year by different authors.

This systematic review included 18 relevant studies, totaling 769 participants. Due to considerable statistical heterogeneity observed across the studies (*I*^2^ = 87.3%, *P* < 0.001), a random-effects model was used to pool the data. The initial analysis indicated that MBT significantly improved upper-limb function after stroke (SMD = 0.90, 95% CI 0.49–1.32, *P* < 0.001; see Figure 6A). To ensure the reliability of the findings, rigorous data diagnostics were performed in accordance with the guidelines in the Cochrane Handbook v6.3. First, Egger's regression test was used to assess potential publication bias caused by small-study effects (52), and no significant publication bias was identified (*t* = 0.098, *P* = 0.923; see Figure 4A). Subsequently, influential studies were identified by combining standardized residuals (|Z| > 2.5) with Cook's distance (> 3 times the mean) (53). Two studies, Ji et al. (45) and Chen and Liu (46), exceeded both thresholds and were therefore excluded (Figure 5). After removing these two outliers, the remaining influential studies were reevaluated using the same combination of standardized residuals and Cook's distance. The results showed that, although the Cook's distances for Wang et al. (48) and Schmid et al. (50) exceeded the threshold, their standardized residuals remained within an acceptable range (see the Supplementary Document). Since neither criterion fully met the exclusion threshold, these studies were retained. Follow-up sensitivity analyses revealed that the overall effect size remained consistent when each study was removed individually, and the trim-and-fill analysis showed that no studies required imputation (see the Supplementary Document for details). Under the robust KHSJ random-effects model, MBT significantly improved upper-limb function in stroke patients compared to the control group (SMD = 0.65, 95% CI 0.37–0.93, *t* = 4.93, *P* < 0.001), with a prediction interval from−0.35 to 1.66 (see the Supplementary Document).

After removing highly influential outliers, the final pooled analysis was conducted on 16 trials. The second Egger's regression test showed no significant publication bias (*t* = −0.320, *P* = 0.753; see Figure 4B). The results confirmed that MBT had a significant moderate-to-large positive impact on upper-limb function recovery in stroke patients (SMD = 0.65, 95% CI 0.40–0.91, *P* < 0.001; refer to Figure 6B). Although some heterogeneity and potential publication bias were detected, the main findings of this study remained statistically robust after thorough validation using Egger's linear regression, sensitivity analysis, the trim-and-fill method, and the KHSJ approach. According to the GRADE assessment, moderate-certainty evidence indicated that traditional Chinese MBT significantly improved upper-limb function in patients with stroke. The certainty of evidence was downgraded by one level due to concerns about the randomization process and outcome measurement in several studies (D1 and D4). However, extensive sensitivity and robustness analyses—including the Knapp–Hartung–Sidik–Jonkman (KHSJ) method, influence diagnostics, the trim-and-fill procedure, and trial sequential analysis—showed that heterogeneity was properly addressed and did not require additional downgrading. No further concerns were identified regarding indirectness, imprecision, or publication bias (see the Supplementary Document for details).

**Figure 7:**
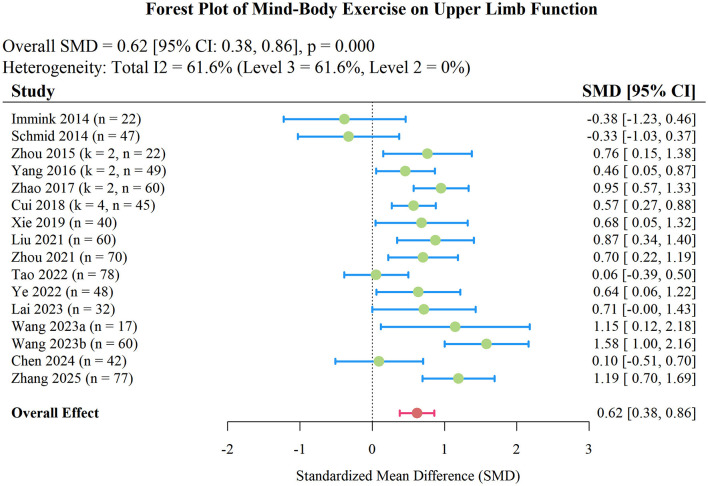
Multilevel forest plot of the effects of mind-body training on upper-limb function in patients with stroke.

To further address the potential for multiple related effect sizes within the same study and to evaluate the robustness of traditional meta-analytic findings, this study conducted a multilevel meta-analysis using the standard random-effects model (Figure 7). The findings showed that MBT had a statistically significant effect on improving upper-limb function in stroke patients (SMD = 0.62, 95% CI 0.38–0.86, *P* = 0.000), with moderate heterogeneity (*I*^2^ = 61.6%). Compared to the results of the traditional meta-analysis (SMD = 0.65, 95% CI 0.40–0.91), the effect size from the multilevel model was slightly smaller; however, the overall direction, statistical significance, and interpretation remained the same. This shows that, after accounting for the correlation among effect sizes within studies, the conclusion that MBT improves upper-limb function in stroke patients remains strong, implying that the main findings are unlikely to be driven solely by violations of the independence assumption.

### Subgroup analysis

**Figure 8:**
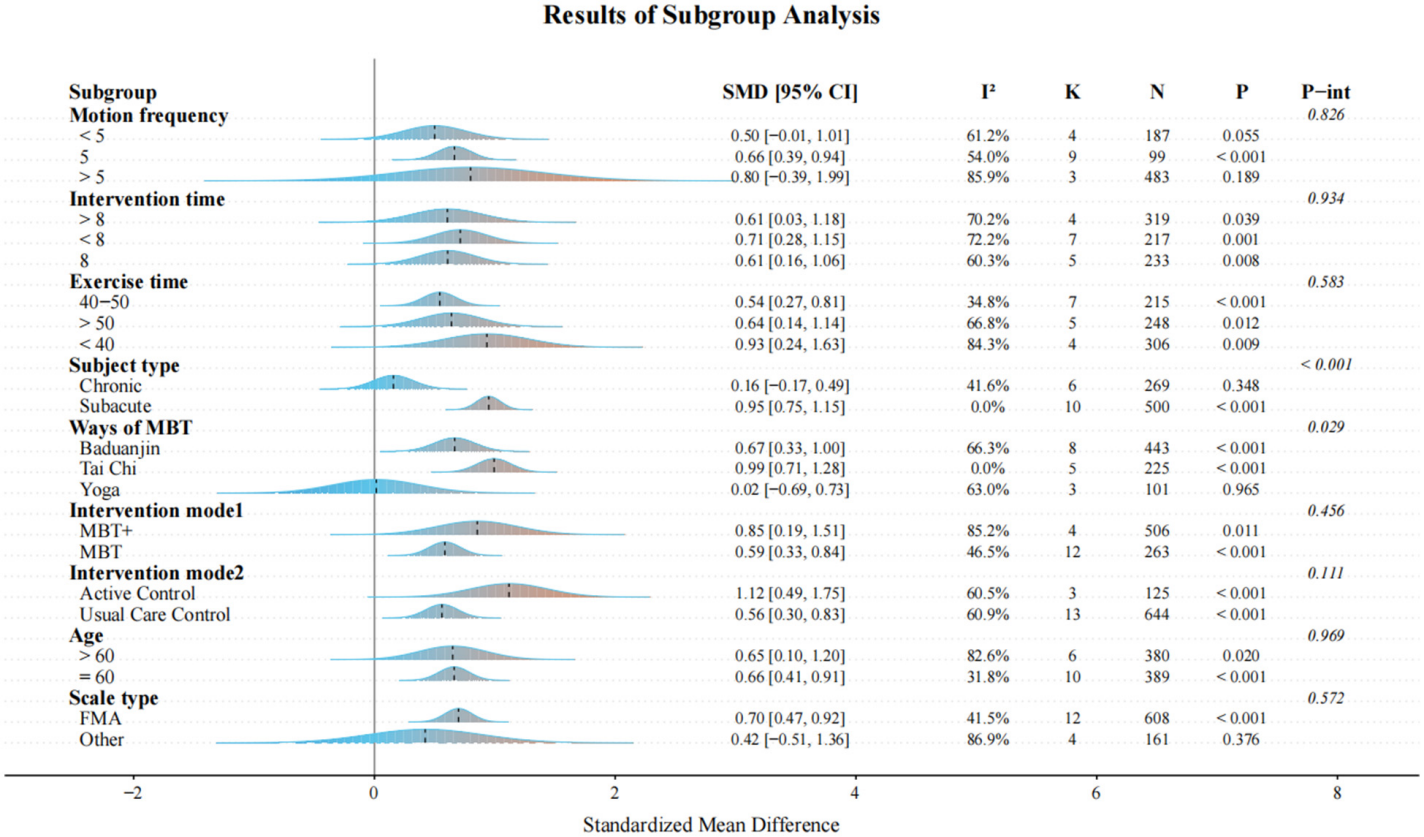
Forest plot of subgroup analyses of the effects of Mind-body training on upper-limb function in patients with stroke. K: Number of studies; N: sample size; P-interaction: interaction tests; MBT: Separate Mind-body training; MBT+: A combined intervention method centered on Mind-body training; Chronic: patients with chronic stroke; Subacute: patients with subacute stroke. Subacute stroke was defined as 7 days to 6 months after stroke onset, whereas chronic stroke was defined as more than 6 months after stroke onset. Disease-stage subgroup results should be interpreted cautiously because some included studies reported the stage category without an exact onset time. Usual Care Control: Standard treatment; Active Control: Other active treatment approaches besides standard treatment; FMA: Fugl-Meyer Assessment score; Other: Other tools for assessing upper-limb function besides the Fugl-Meyer Assessment.

To further explore the sources of heterogeneity and the main moderating factors affecting MBT's effects on upper-limb function in stroke patients, this study employed a systematic subgroup analysis. The analysis included the following prespecified variables: (1) intervention frequency; (2) intervention duration; (3) session length; (4) patient type; (5) type of MBT; (6) intervention mode; (7) control-group intervention mode; (8) patient age; and (9) measurement tool. The subgroup analysis confirmed that the effects of MBT were inconsistent but were significantly influenced by the interaction between the intervention design and patients' clinical characteristics (see Figure 8 for details).

Regarding disease stage, the interaction test revealed significant differences in treatment effects across disease stages (*P* < 0.001). Patients in the subacute stroke stage experienced greater improvement after MBT (*K* = 10, SMD = 0.95, *P* < 0.001), while those in the chronic stage showed only a small, nonsignificant improvement (*K* = 6, SMD = 0.16, *P* = 0.348). Regarding the training modality, the extent of improvement varied significantly across MBT types (P-int = 0.029). Tai Chi resulted in the most notable improvement in upper-limb function among stroke patients (*K* = 5, SMD = 0.99, *P* < 0.001), followed by Baduanjin, which also demonstrated a meaningful moderate-to-large effect (*K* = 8, SMD = 0.67, *P* < 0.001). In contrast, yoga did not show a significant therapeutic effect in the samples analyzed in this study (*K* = 3, SMD = 0.02, *P* = 0.965).

Regarding intervention parameters, the most positive effects were observed with five sessions per week (*K* = 9, SMD = 0.66, *P* < 0.001), an intervention lasting less than 8 weeks (*K* = 7, SMD = 0.71, *P* = 0.001), and each session lasting under 40 min (*K* = 4, SMD = 0.93, *P* = 0.009). This suggests that short-duration, high-frequency stimulation might be more effective for upper-limb functional recovery than extended low-intensity training. Regarding intervention strategies, combined approaches that include MBT as a core component, along with other conventional rehabilitation methods, result in greater improvements in upper-limb function for stroke patients (*K* = 4, SMD = 0.85, *P* = 0.011) compared to MBT alone (*K* = 12, SMD = 0.59, *P* < 0.001). Even when the control group received active interventions, the MBT group continued to demonstrate significant superiority (*K* = 3, SMD = 1.12, *P* < 0.001). Regarding age, patients aged 60 years or younger experienced greater benefits (*K* = 10, SMD = 0.66, *P* < 0.001). Regarding the sensitivity of outcome measures, the FMA showed a stronger ability to detect effects (*K* = 12, SMD = 0.70, *P* < 0.001), indicating significantly greater sensitivity than other assessment tools (*K* = 4, SMD = 0.42, *P* = 0.376).

### Linear meta-regression

**Figure 9:**
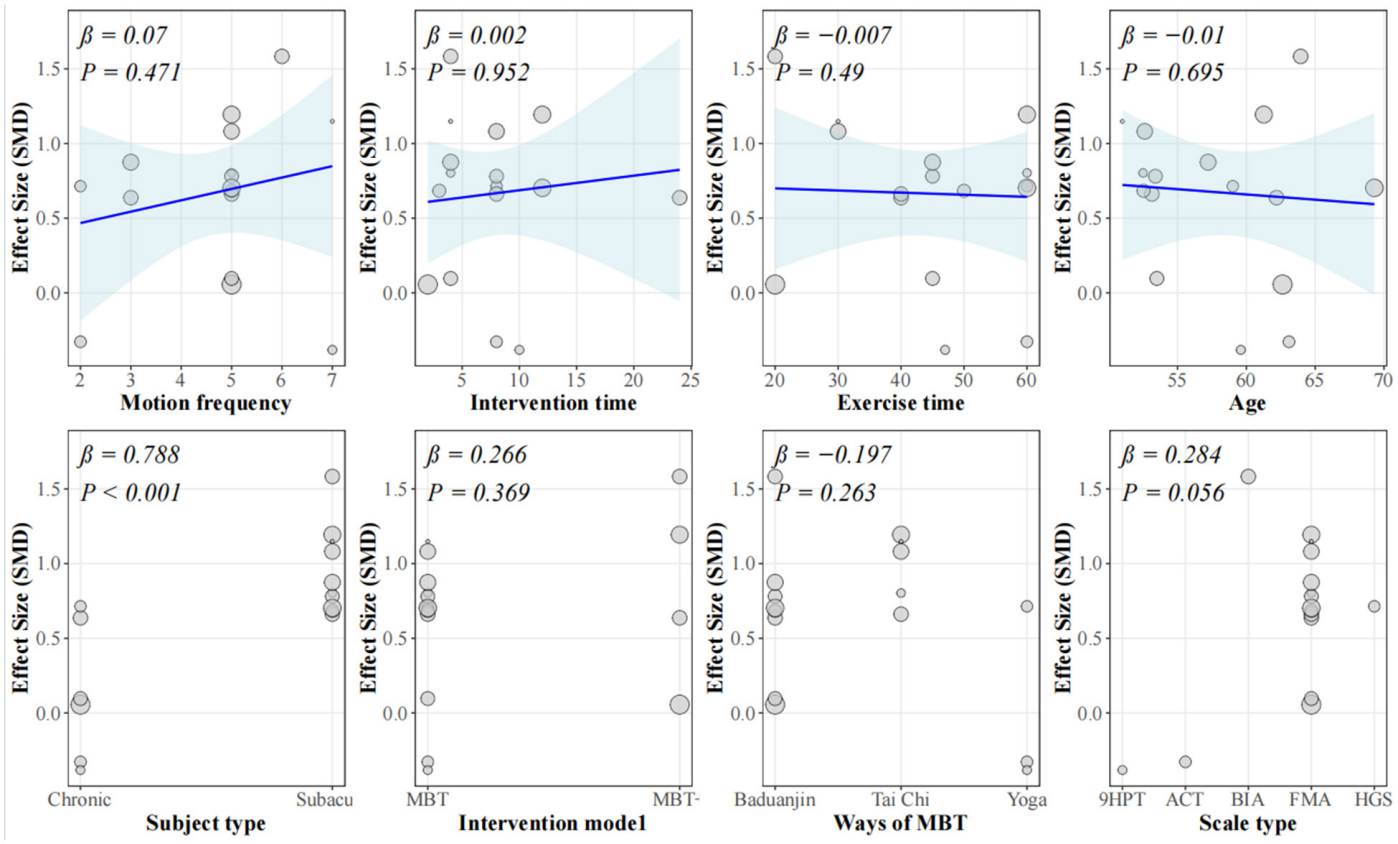
Linear meta-regression bubble plot of the effects of Mind-body training on upper-limb function in patients with stroke. The solid line represents the fitted linear meta-regression line. The light blue shaded area indicates the 95% confidence interval (CI) around the predicted mean effect. The bubbles represent the estimated effect sizes from individual studies.

To better clarify potential confounding factors and identify independent predictors of treatment effectiveness, this study conducted a multivariable meta-regression analysis (see Figure 9 for details). The model revealed that patient type was the only independent variable that significantly predicted the degree of improvement in upper-limb function (β = 0.788, *p* < 0.001). Since this analysis was a study-level secondary analysis rather than an individual patient data analysis, there is a potential risk of ecological fallacy, and the direction of the SMD depends heavily on how the original data's variables were coded. Therefore, the regression results of this study should mainly be viewed as initial indicators of trends and interpreted with caution. To obtain more definitive causal conclusions, future research should conduct large-scale RCTs or perform more detailed validation using pooled individual patient data analyses.

### Nonlinear dose–response analysis

**Figure 10:**
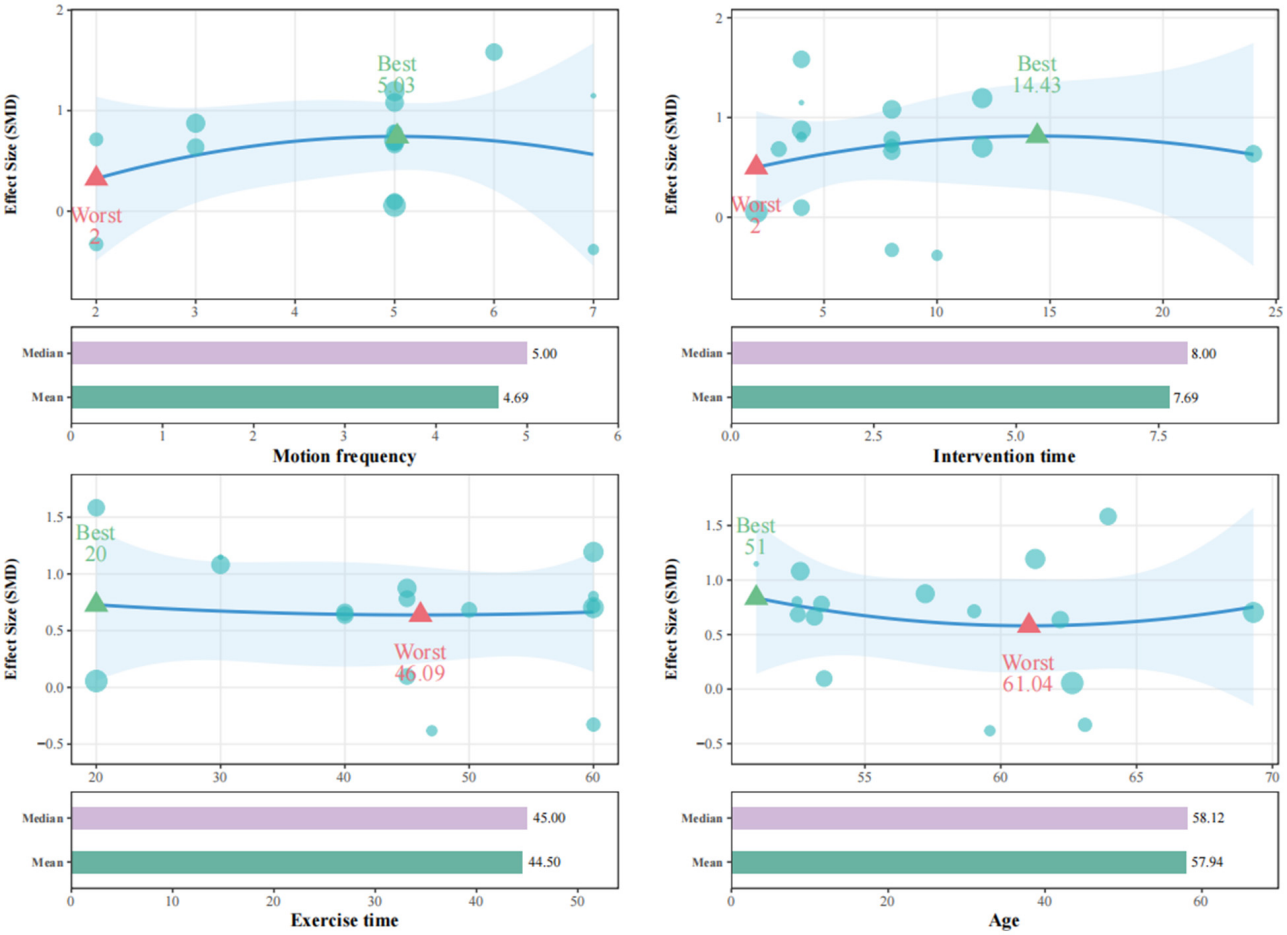
Nonlinear regression plot of the effects of mind-body training on upper-limb function in patients with stroke.

**Figure 11:**
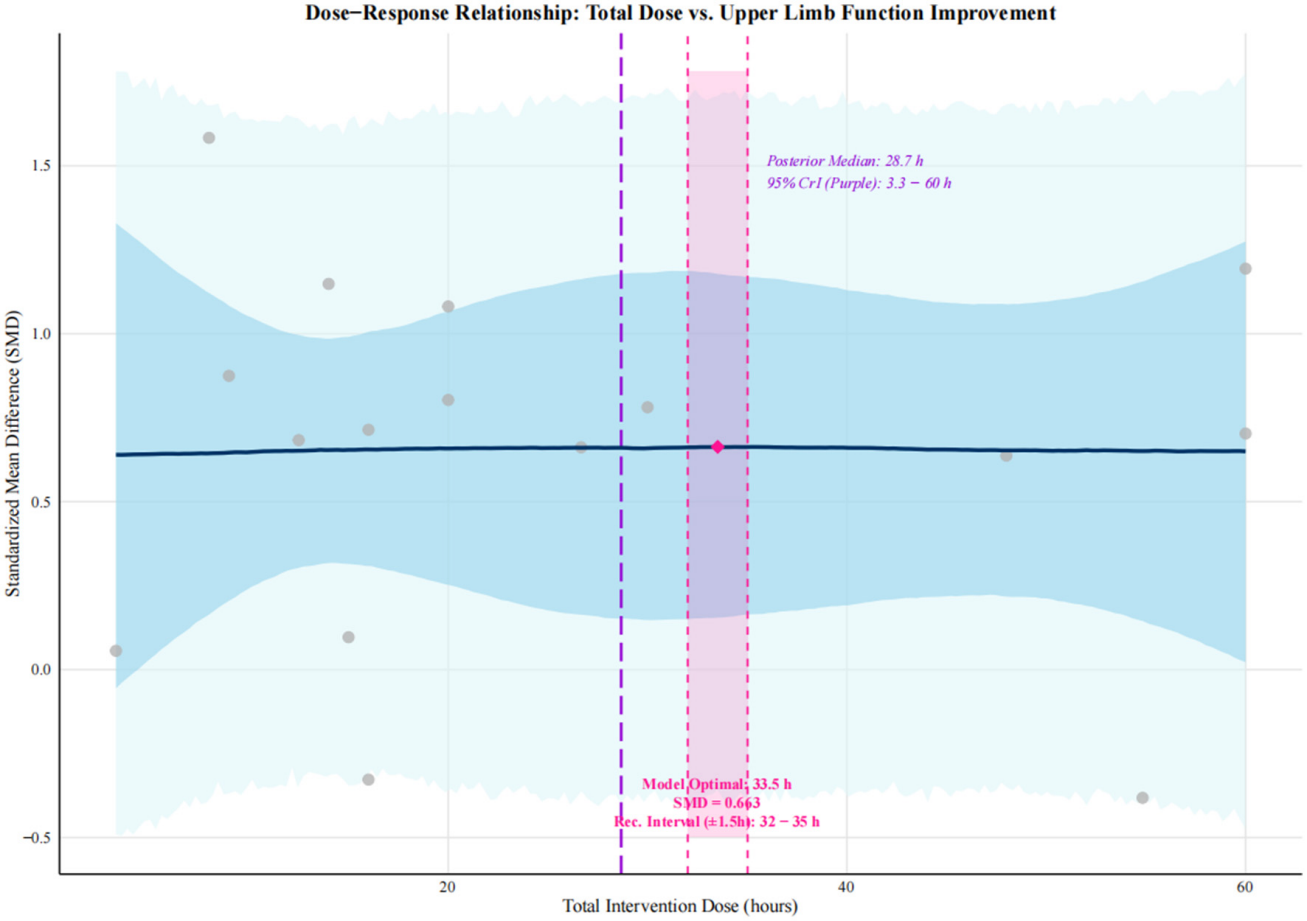
Dose-response relationship plot of the effects of mind-body training on upper-limb function in patients with stroke.

The nonlinear meta-regression analysis revealed that improvements in upper-limb function in stroke patients following MBT do not follow a simple linear pattern; instead, they exhibit a clear nonlinear trend characterized by an inverted U shape. This suggests a threshold for diminishing marginal benefits, indicating that moderate doses and frequencies of intervention are more effective than extreme levels. Based on the quadratic model fitting and the sample density distribution (see Figure 10), several key parameter characteristics were identified. Regarding frequency, the fitted curve indicated an optimal intervention frequency of about 5 sessions per week, which closely matches the common settings used in current studies (median: 5 sessions/week; mean: 4.69 sessions/week), confirming the validity of the current mainstream clinical frequency. The model predicts an optimal intervention duration of about 14 weeks, whereas most existing empirical studies have used shorter treatment durations (mean: 7.69 weeks; median: 8 weeks). This indicates that, in clinical practice, extending the intervention duration to approximately 14 weeks might yield greater functional improvements. The model-estimated ideal session length is about 20 min; however, because of the wide confidence interval around this estimate and its location within an area with limited original data points, its stability and applicability are limited. Therefore, this finding should be viewed as preliminary evidence that shorter session durations may be more effective than very long ones, rather than treating 20 min as the only ideal length. Considering the typical intervention duration range in current studies and practical factors in clinical training settings, this study also supports a 30-min duration as a more reliable and feasible compromise. Based on the overall analysis of the Bayesian dose-response curve (see Figure 11), the optimal estimated cumulative intervention duration was approximately 33.5 h (SMD ≈ 0.66), with the recommended effective range being 32–35 h. Notably, the confidence intervals at both ends of the curve widened considerably, indicating increased uncertainty in the effect estimates at extreme doses.

In summary, we recommend an evidence-based ‘moderate prescription' with a suggested frequency of 5 sessions per week, each lasting about 30 min, over 14–15 weeks. Although the total duration of this regimen exceeds the model-predicted optimal dose (around 32–35 h), this combination effectively balances the performance of each parameter. Additionally, this study provides the posterior distribution of the model-estimated optimal dose, along with the global density overlay plot and the posterior predictive interval plot (see the Supplementary Document for details). This conclusion is based on a secondary, non-randomized regression analysis, which is limited by differences in variable coding across the original studies, uneven sample distributions, and heterogeneity. Additionally, some optimal parameters were identified in data-sparse regions, requiring flexible adjustments in clinical applications. Further validation through large-sample RCTs or individual participant data analyses remains necessary.

### XGBoost machine learning exploratory analysis

**Figure 12:**
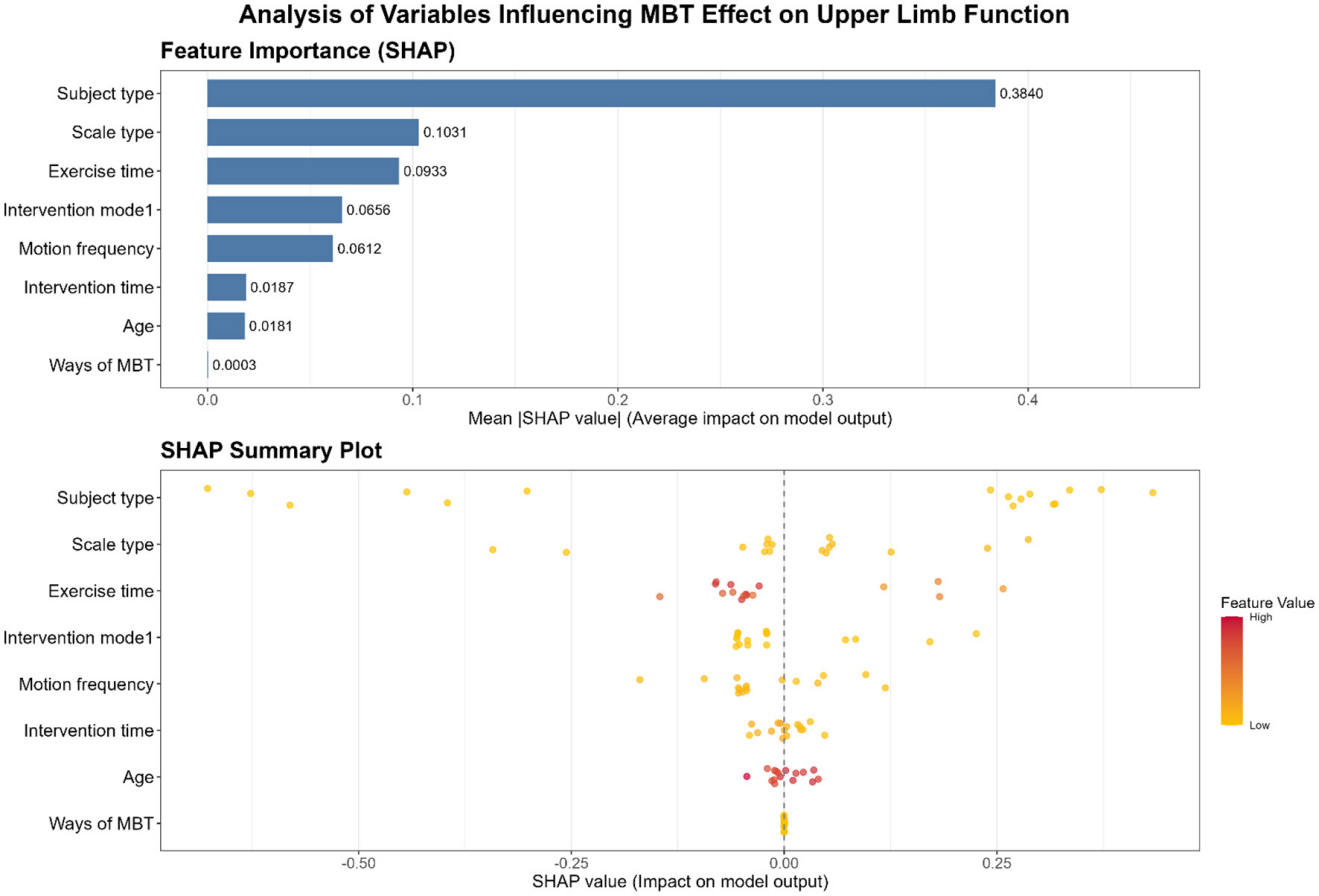
SHAP bar plot and beeswarm plot of the effects of mind-body training on upper-limb function in patients with stroke. *Note:* Exercise time = single session duration (minutes). Motion frequency = sessions per week. Intervention time = total intervention duration. Stroke stage: Chronic stroke = patients with chronic stroke; Subacute stroke = patients with subacute stroke; Acute stroke = patients with acute stroke. Intervention mode: MBT, mind-body training alone; MBT+, mind-body training combined with conventional rehabilitation or another co-intervention. MBT modality refers to the type of mind-body training, including Tai Chi, Baduanjin, and yoga.

According to the SHAP feature importance ranking (upper panel of Figure 12), the stroke stage was identified as the most important variable for predicting improvements in upper-limb function among stroke patients undergoing MBT, with a mean absolute SHAP value (|SHAP| ≈ 0.38) notably surpassing that of other factors. The second tier of influential variables included outcome measure type and session duration, both with SHAP values of 0.09–0.10. In contrast, intervention mode and frequency contributed moderately, with SHAP values of 0.06 and 0.07, respectively. Meanwhile, total intervention duration and patient age had relatively low predictive importance (|SHAP| ≈ 0.02), and the specific type of MBT provided minimal additional value to the model predictions (|SHAP| ≈ 0).

Further examination of the SHAP summary plot (lower panel of Figure 12, beeswarm plot) revealed a clear left-right separation of sample points based on subject type along the SHAP axis. This pattern shows that different patient subgroups significantly influence the assessment of treatment effectiveness, characterized by a complex nonlinear relationship rather than a simple linear trend. Overall, the model indicates that patients' pathological features should be the primary focus when assessing MBT's effectiveness. However, due to uneven sample distribution across predictor variables and potential heterogeneity, these results should be interpreted carefully and in context with the study design. The associated learning curves and detailed SHAP data files are available in the Supplementary Document.

## Summary

**Figure 13:**
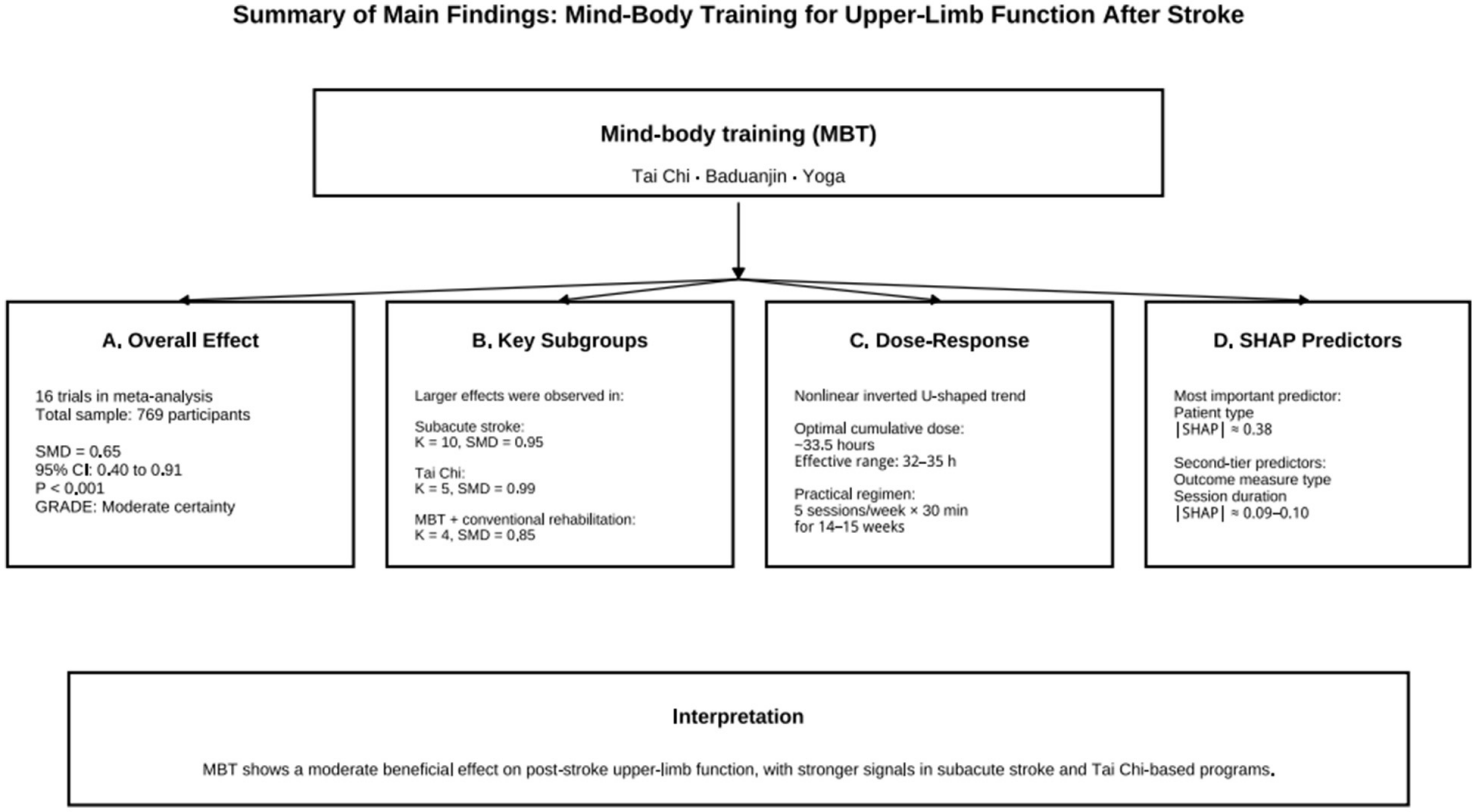
Summary of the main findings. Subgroup, dose response, and SHAP findings are exploratory and based on study-level data; they should be interpreted cautiously and validated in future dose-stratified RCTs.

The pooled analysis confirmed that MBT significantly improved upper-limb motor function in stroke patients, demonstrating a moderate positive effect (SMD = 0.65, *P* < 0.001). Despite unavoidable heterogeneity and potential publication bias across the original studies, the main conclusion remained reliable after thorough checks with Egger's test, the trim-and-fill method, sensitivity analysis, and screening for highly influential studies. Furthermore, subgroup and nonlinear dose-response analyses showed that the intervention effect did not increase linearly but tended to peak in the moderate-to-high dose range (recommended regimen: 5 sessions per week, about 30 min per session, for 14–15 weeks). Additionally, the SHAP analysis identified patient type as the most important predictor of prognosis, with the highly variable distribution of its sample values further supporting significant individual-level and between-study differences. Overall, the existing evidence supports MBT as an effective rehabilitation method with moderate therapeutic benefits, while emphasizing the importance of considering individual differences and optimizing dosage. To enhance the clarity and clinical interpretability of our findings, we have included a summary figure (Figure 13) that integrates the overall pooled effect, key subgroup findings, study-level dose-response results, and SHAP-based predictor ranking.

## Discussion

This study demonstrated that MBT significantly improved upper-limb function in patients with stroke. After excluding highly influential studies, the overall effect size across 16 trials was SMD = 0.65 (95% CI 0.40–0.91, *P* < 0.001), with consistent results from the robust KHSJ model. After accounting for within-study effect-size dependence through a multilevel meta-analysis, the effect size slightly decreased to SMD = 0.62 (95% CI 0.38–0.86); however, the direction of the effect and the statistical conclusion remained the same, indicating that the main findings of this study are reliable. Although heterogeneity was high in the initial analysis, the available evidence still supports MBT as an effective adjunctive strategy for upper-limb rehabilitation after stroke, especially when considering influence diagnostics, sensitivity analyses, Egger's test, and trim-and-fill results collectively. Consistent with previous reviews, MBT, such as Tai Chi, Baduanjin, and yoga, generally helps improve physical function and rehabilitation outcomes in stroke patients. However, unlike previous studies that mainly focused on walking, balance, or overall function, this study specifically combined outcomes related to independent upper-limb function. As a result, it more directly addressed the functional area most noticeably limited in the daily lives of stroke patients and better demonstrated the potential additional benefit of MBT in upper-limb rehabilitation (54, 55). Furthermore, Van de Winckel et al. (56) demonstrated that cognitive multisensory rehabilitation training improved upper-limb motor function and enhanced activities of daily living related to social participation in adults with chronic stroke. This finding aligns with the present study's results (56).

Subgroup analyses further revealed that differences in treatment effects were mainly attributable to the disease stage and the method of intervention implementation. Patients in the subacute phase experienced greater benefits, whereas those in the chronic phase showed smaller effects that were not statistically significant. This finding generally supports the stroke recovery staging theory, indicating that the subacute phase is a crucial period characterized by increased neuroplasticity and responsiveness to training, during which patients are more likely to benefit from structured rehabilitation interventions (26, 55). At the same time, patient type was the only significant independent predictor in the meta-regression, and the XGBoost-SHAP analysis also identified it as the most important variable. This suggests that disease stage is likely the main factor explaining the differences in effect sizes across studies. However, this does not mean that patients in the chronic phase did not benefit; rather, it suggests that studies involving patients in the chronic phase often had smaller sample sizes, greater variability in disease progression, and more pronounced differences in baseline functioning. Therefore, this finding should be viewed as an indicator of the subgroup most likely to benefit, rather than a definitive stratified conclusion. Regarding training modality, both Tai Chi and Baduanjin showed clear benefits, whereas yoga did not. MBT combined with conventional rehabilitation was more effective than MBT alone, with additional benefits still observed even under active control conditions. This suggests that MBT is more likely to serve as an additive component within current upper-limb rehabilitation systems rather than as a replacement method (57). Consistent with the task-oriented principles emphasized in earlier rehabilitation research—namely, high repetition, clear goals, and transferability to daily activities—the better outcomes of Tai Chi and Baduanjin may be attributed not only to their training techniques but also to their steady rhythm, repetitive movements, bilateral engagement, and easier incorporation into routine rehab programs (58–60).

Another key finding of this study is that the relationship between MBT and upper-limb recovery is not linear; rather, it exhibits an inverted-U-shaped dose-response curve. The nonlinear analysis shows that the optimal frequency is approximately five sessions per week, the ideal intervention duration is around 14 weeks, and the most effective total intervention time is roughly 33.5 h, with an effective range from 32 to 35 h. Although the model estimates the optimal session duration at around 20 min, data within this range are somewhat limited, leading to considerable uncertainty. Given the common intervention settings in existing studies and the practicality of clinical implementation, a 30-min session duration may offer a more reliable compromise. Based on this, the study recommends a “moderate-dose” protocol of five sessions per week, each approximately 30 min, for 14–15 weeks. The subgroup analysis shows significant effects within 8 weeks, while the continuous-dose model indicates the optimal duration is around 14 weeks; these results are not at odds with each other. The first suggests that short-term interventions can be effective, while the second reflects the overall trend of cumulative training stimulus and diminishing returns. Since frequency, session duration, and intervention period are interconnected, and some optimal points are in data-sparse areas, the current dose-related conclusions should be viewed as directional guidance for prescription optimization rather than the absolute best regimen. This aligns with the principle outlined in stroke rehabilitation guidelines, which state that prescriptions should be sufficient, repetitive, and personalized (55, 61). At this stage, a more practical clinical approach is to use MBT as a feasible complement to post-stroke upper-limb rehabilitation, focusing on patients in the subacute phase. Its application should be tailored to conventional rehabilitation, taking into account patient tolerance, training goals, and resource availability. Future dose-stratified RCTs or individual participant data meta-analyses are still needed to further confirm the optimal protocol and its feasible limits.

## Conclusion

The meta-analysis in this study found that MBT has a significant moderate positive impact on upper-limb motor function in stroke patients (SMD = 0.65). A “moderate-dose” treatment plan, consisting of five sessions per week for about 30 min each over 14–15 weeks, is recommended to maximize marginal benefits. In clinical practice, MBT can be an essential part of comprehensive stroke rehabilitation, especially for patients in the subacute phase. Furthermore, Tai Chi or combined rehabilitation approaches have been shown to produce more substantial effects. The XGBoost-SHAP analysis identified patient type as the most important predictor of treatment effectiveness, followed by outcome measures and session length, thereby providing crucial evidence to explain the variability in treatment effects observed in previous studies. Overall, this study suggests that individualized MBT prescriptions should be tailored to the patient's disease stage, and larger-scale RCTs or analyses of individual participant data are necessary to further verify model-estimated optimal dose parameters and their long-term stability.

## Limitations

This study has several limitations. First, the initial pooled analysis revealed high heterogeneity (*I*^2^ = 87.3%), and the prediction interval ranged from −0.35 to 1.66, indicating that the true effect size could have varied substantially across study settings. Second, the main sources of bias were primarily found in the randomization process (D1) and outcome measurement (D4). Some studies provided insufficient information about how missing outcome data (D3) were handled, which could have impacted the accuracy of effect estimates. Third, most of the included studies were conducted in China, with only a small portion of data from the United States and Australia, limiting the generalizability of the findings. Fourth, the outcome data in this study were collected at the immediate post-intervention time point, and there remains a lack of direct evidence on whether the long-term effects and apparent benefits observed in the subacute phase can be sustained. Fifth, the meta-regression and dose-response analyses were conducted at the study level rather than using individual participant data, which raises the risk of ecological fallacy. Furthermore, the direction of the standardized mean difference depended on the variable coding. Furthermore, some of the best parameter estimates were derived from regions with relatively few original data points, and the signals related to intervention duration were not entirely consistent between the categorical subgroup analysis and the continuous dose model. Therefore, the current dose recommendations should be viewed more as initial guidance for prescription optimization rather than as definitive optimal regimens.

## Data Availability

The original contributions presented in the study are included in the article/Supplementary material, further inquiries can be directed to the corresponding author.

## References

[1] Feigin VL Abate MD Abate YH Abd ElHafeez S Abd-Allah F Abdelalim A . Global, regional, and national burden of stroke and its risk factors, 1990–2021: a systematic analysis for the global burden of disease study 2021. Lancet Neurol. (2024) 23:973–1003. doi: 10.1016/S1474-4422(24)00369-7 39304265 PMC12254192

[2] Qin C Yang S Chu YH Zhang H Pang XW Chen L . Signaling pathways involved in ischemic stroke: molecular mechanisms and therapeutic interventions. Sig Transduct Target Ther. (2022) 7:215. doi: 10.1038/s41392-022-01064-1 35794095 PMC9259607

[3] Purrahman D Shojaeian A Poniatowski ŁA Piechowski-Józwiak B Mahmoudian-Sani MR. The role of progranulin (PGRN) in the pathogenesis of ischemic stroke. Cell Mol Neurobiol. (2023) 43:3435–47. doi: 10.1007/s10571-023-01396-8 37561339 PMC11410000

[4] Mayo NE Wood-Dauphinee S Côté R Durcan L Carlton J. Activity, participation, and quality of life 6 months poststroke. Arch Phys Med Rehabil. (2002) 83:1035–42. doi: 10.1053/apmr.2002.33984 12161823

[5] Langhorne P Bernhardt J Kwakkel G. Stroke rehabilitation. Lancet. (2011) 377:1693–702. doi: 10.1016/S0140-6736(11)60325-5 21571152

[6] Levine DA Galecki AT Langa KM Unverzagt FW Kabeto MU Giordani B . Trajectory of cognitive decline after incident stroke. Available online at: https://jamanetwork.com/journals/jama/fullarticle/2382979 (Accessed February 17, 2026). 10.1001/jama.2015.6968 PMC4655087 26151265

[7] Ankolekar S Geeganage C Anderton P Hogg C Bath PMW. Clinical trials for preventing post stroke cognitive impairment. J Neurol Sci. (2010) 299:168–74. doi: 10.1016/j.jns.2010.08.052 20855090

[8] Tatemichi TK Paik M Bagiella E Desmond DW Pirro M Hanzawa LK. Dementia after stroke is a predictor of long-term survival. Stroke. 1994. 25:1915–1919. doi: 10.1161/01.STR.25.10.1915 8091433

[9] Saunders DH Greig CA Mead GE. Physical activity and exercise after stroke. Stroke. (2014) 45:3742–7. doi: 10.1161/STROKEAHA.114.004311 25370588

[10] Saunders DH Sanderson M Hayes S Johnson L Kramer S Carter DD . Physical fitness training for stroke patients. Cochrane Database Syst Rev. (2020) 3:CD003316. doi: 10.1002/14651858.CD003316.pub7 32196635 PMC7083515

[11] Miller KK Porter RE DeBaun-Sprague E Puymbroeck MV Schmid AA. Exercise after stroke: patient adherence and beliefs after discharge from rehabilitation. Topics in Stroke Rehabilitation. 2017. Located at: world. doi: 10.1080/10749357.2016.1200292 27334684

[12] Koh TC. Baduanjin – an ancient Chinese exercise. Am J Chin Med. (1982) 10:14–21. doi: 10.1142/S0192415X8200004X 7183203

[13] Jiang Y Zou J. Analysis of the TCM theory of traditional Chinese health exercise. J Sport Health Sci. (2013) 2:204–8. doi: 10.1016/j.jshs.2013.03.008

[14] Li M Fang Q Li J Zheng X Tao J Yan X . The effect of Chinese traditional exercise-baduanjin on physical and psychological well-being of college students: a randomized controlled trial. PLoS One. (2015) 10:e0130544. doi: 10.1371/journal.pone.0130544 26158769 PMC4497728

[15] Zou L Pan Z Yeung A Talwar S Wang C Liu Y . A review study on the beneficial effects of baduanjin. J Altern Complement Med Paradigm, Practice, and Policy Advancing Integrative Health. (2018). Located at: Sage CA: Los Angeles, CA. Available online at: https://journals.sagepub.com/doi/full/10.1089/acm.2017.0241 10.1089/acm.2017.0241 29227709

[16] Zou L Loprinzi PD Yeung AS Zeng N Huang T. The beneficial effects of mind-body exercises for people with mild cognitive impairment: a systematic review with meta-analysis. Arch Phys Med Rehabil. (2019) 100:1556–73. doi: 10.1016/j.apmr.2019.03.009 30986409

[17] Zou L Wang C Chen X Wang H. Baduanjin exercise for stroke rehabilitation: a systematic review with meta-analysis of randomized controlled trials. Int J Environ Res Public Health. (2018) 15:600. doi: 10.3390/ijerph15040600 29584623 PMC5923642

[18] Zheng G Zheng Y Xiong Z Ye B. Effect of baduanjin exercise on cognitive function in patients with post-stroke cognitive impairment: a randomized controlled trial. Clin Rehabil. (2020) 34:1028–39. doi: 10.1177/0269215520930256 32517490

[19] Lin IPH Miao JH Ching FS Su L Ching LH Jau H. Psychometric comparisons of 2 versions of the fugl-meyer motor scale and 2 versions of the stroke rehabilitation assessment of movement. Neurorehabilit Neural Repair. (2008). Available online at: https://journals.sagepub.com/doi/abs/10.1177/1545968308315999 10.1177/1545968308315999 18645189

[20] Sanford J Moreland J Swanson LR Stratford PW Gowland C. Reliability of the fugl-meyer assessment for testing motor performance in patients following stroke. Phys Ther. (1993) 73:447–54. doi: 10.1093/ptj/73.7.447 8316578

[21] Moher D Shamseer L Clarke M Ghersi D Liberati A Petticrew M . Preferred reporting items for systematic review and meta-analysis protocols (PRISMA-P) 2015 statement. Syst Rev. (2015) 4:1. doi: 10.1186/2046-4053-4-1 25554246 PMC4320440

[22] Nakagawa S Cuthill IC. Effect size, confidence interval and statistical significance: a practical guide for biologists. Biol Rev. (2007) 82:591–605. doi: 10.1111/j.1469-185X.2007.00027.x 17944619

[23] Lau J Ioannidis JPA Schmid CH. Quantitative synthesis in systematic reviews. Ann Intern Med. (1997) 127:820–6. doi: 10.7326/0003-4819-127-9-199711010-00008 9382404

[24] Viechtbauer W Cheung MW. Outlier and influence diagnostics for meta-analysis. Res Synth Methods. (2010) 1:112–25. doi: 10.1002/jrsm.11 26061377

[25] Van Houwelingen HC Arends LR Stijnen T. Advanced methods in meta-analysis: multivariate approach and meta-regression. Stat Med. (2002) 21:589–624. doi: 10.1002/sim.1040 11836738

[26] Bernhardt J Hayward KS Kwakkel G Ward NS Wolf SL Borschmann K . Agreed definitions and a shared vision for new standards in stroke recovery research: the stroke recovery and rehabilitation roundtable taskforce. Int J Stroke. (2017) 12:444–50. doi: 10.1177/1747493017711816 28697708

[27] Hedges LV Olkin I. Statistical Methods for Meta-Analysis. New York, NY: Academic Press. (2014).

[28] Crippa A Orsini N. Multivariate dose-response meta-analysis: the dosresmeta R package. J Stat Softw. (2016) 72:1–15. doi: 10.18637/jss.v072.c01

[29] Hamza T Cipriani A Furukawa TA Egger M Orsini N Salanti G . Bayesian dose–response meta-analysis model: a simulations study and application. Stat Methods Med Res. (2021) 30:1358–72. doi: 10.1177/0962280220982643 33504274 PMC8209313

[30] Van den Noortgate W López-López JA Marín-Martínez F Sánchez-Meca J. Three-level meta-analysis of dependent effect sizes. Behav Res. (2013) 45:576–94. doi: 10.3758/s13428-012-0261-6 23055166

[31] Wang H Liang Q Hancock JT Khoshgoftaar TM. Feature selection strategies: a comparative analysis of SHAP-value and importance-based methods. J Big Data. (2024) 11:44. doi: 10.1186/s40537-024-00905-w

[32] Chen T Guestrin C. Xgboost: A Scalable Tree Boosting System New York, NY: Association for Computing Machinery (ACM). (2016). p. 785–94. doi: 10.1145/2939672.2939785

[33] Sterne JA Savović J Page MJ Elbers RG Blencowe NS Boutron I . RoB 2: a revised tool for assessing risk of bias in randomised trials. BMJ. (2019) 366:14898. doi: 10.1136/bmj.l4898 31462531

[34] Ye M Zheng Y Xiong Z Ye B Zheng G. Baduanjin exercise ameliorates motor function in patients with post-stroke cognitive impairment: a randomized controlled trial. Complement Ther Clin Pract. (2022) 46:101506. doi: 10.1016/j.ctcp.2021.101506 34742096

[35] Zhang L Yu X Liao W Wang J Lu Y Wang N . Effects of body weight-supported tai chi yunshou training on upper limb motor function in stroke patients: A three-arm parallel randomized controlled trial. PLoS One. (2025) 20:e0314025. doi: 10.1371/journal.pone.0314025 39787119 PMC11717223

[36] Tao J Zhang S Kong L Zhu Q Yao C Guo Q . Effectiveness and functional magnetic resonance imaging outcomes of tuina therapy in patients with post-stroke depression: a randomized controlled trial. Front Psychiatr. (2022) 13:923721. doi: 10.3389/fpsyt.2022.923721 35845459 PMC9281445

[37] Lai YT Huang HL Hsieh CC Lin CH Yang JC Tsou HH . The effects of yoga exercise on blood pressure and hand grip strength in chronic stroke patients: a pilot controlled study. Int J Environ Res Public Health. (2023) 20:1108. doi: 10.3390/ijerph20021108 36673861 PMC9859542

[38] Yang HX Tang Q. Clinical observation of Tai Chi for rehabilitation of motor dysfunction in stroke patients. Chinese J Rehabilit Med. (2016) 31:1146–8.

[39] Zhao B Tang Q Wang Y Zhu LW Yang HX Ye T. Effects of Tai Chi on motor function and depressive state in patients with post-stroke depression. Chinese J Rehabilit Theory Pract. (2017) 23:334–7. doi: 10.3969/j.issn.1006-9771.2017.03.019

[40] Zhou L Li ZH Zhang Y Chen K Lin Y. Preliminary study on the rehabilitative efficacy of modified Tai Chi for motor function in stroke patients. Chinese J Integrat Med Cardio-/Cerebrovascular Dis. (2015) 13:878–80.

[41] Cui YS Wang MJ Yang HX. Analysis of the effects of Health Qigong Baduanjin on motor function in patients during the recovery phase of stroke. J Shandong Instit Phys Educ Sports. (2018) 34:97–100.

[42] Xie BJ Yang M Bai YL. Clinical study on the effect of Baduanjin on motor function recovery in stroke patients. West China Med J. (2019) 34:515–9.

[43] Liu XY Zeng WJ Liu J Ai X Deng GR. Effects of traditional Baduanjin exercise on post-stroke depression. Clin J Chinese Med. (2021) 13:86–8.

[44] Zhou HY Wu YY Wu CL. Effects of Baduanjin combined with rehabilitation training in elderly stroke patients during the recovery phase. Geriatr Health Care. (2021) 27:1191–4.

[45] Ji XY Yuan M Song Y. Effects of Baduanjin intervention on motor function, electromyographic characteristics, and serum neurocytokine levels in stroke patients with hemiplegia. Clin Res Pract. (2022) 7:18–22.

[46] Chen Q Liu HP. Observation on the rehabilitation effect of sitting Baduanjin combined with modern rehabilitation training on upper-limb dysfunction in post-stroke hemiplegic patients. Guide China Med. (2023) 21:32–5.

[47] Wang WH Zhang GP Xie HJ Qu PY Qu PC Wang XH . Effect of sitting Tai Chi on upper-limb motor function in Brunnstrom stage II stroke patients. J Chengdu Sport Univ. (2023a) 49:82–7. doi: 10.15942/j.jcsu.2023.02.012

[48] Wang YZ Wen QX Li XX. Effects of Baduanjin combined with action observation therapy on muscle strength in stroke patients with hemiplegia. Heilongjiang J Trad Chinese Med. (2023b) 52:169–71.

[49] Chen JW Chen Q Chen C Li SY Liu LL Wu CS . Effect of modified Baduanjin exercise on cardiopulmonary function, motor function and activities of daily living for stroke patients. Chinese J Rehabilit Theory Pract. (2024) 30:74–80.

[50] Schmid AA Miller KK Van Puymbroeck M DeBaun-Sprague E. Yoga leads to multiple physical improvements after stroke, a pilot study. Complement Ther Med. (2014) 22:994–1000. doi: 10.1016/j.ctim.2014.09.005 25453519

[51] Immink MA Hillier S Petkov J. Randomized controlled trial of yoga for chronic poststroke hemiparesis: Motor function, mental health, and quality of life outcomes. Top Stroke Rehabil. (2014) 21:256–71. doi: 10.1310/tscir2101-256 24985393

[52] Egger M Smith GD Schneider M Minder C. Bias in meta-analysis detected by a simple, graphical test. BMJ. (1997) 315:629–34. doi: 10.1136/bmj.315.7109.629 9310563 PMC2127453

[53] Viechtbauer W Cheung MWL. Outlier and influence diagnostics for meta-analysis. Res Synth Methods. (2010) 1:112–25. doi: 10.1002/jrsm.11 26061377

[54] Dong J Chi J Wang D. Effects of mind–body exercise on physical ability, mental health and quality of life in stroke patients: a systematic review and meta-analysis. Front Public Health. (2024) 12:1432510. doi: 10.3389/fpubh.2024.1432510 39758196 PMC11697286

[55] Winstein CJ Stein J Arena R Bates B Cherney LR Cramer SC . Guidelines for adult stroke rehabilitation and recovery. Stroke. (2016) 47:e98–169. doi: 10.1161/STR.0000000000000098 27145936

[56] Van De Winckel A De Patre D Rigoni M Fiecas M Hendrickson TJ Larson M . Exploratory study of how cognitive multisensory rehabilitation restores parietal operculum connectivity and improves upper limb movements in chronic stroke. Sci Rep. (2020) 10:20278. doi: 10.1038/s41598-020-77272-y 33219267 PMC7680110

[57] Barclay RE Stevenson TJ Poluha W Semenko B Schubert J. Mental Practice for Treating Upper Extremity Deficits in Individuals with Hemiparesis after Stroke. Cochrane Library. (2020). Available online at: https://www.cochranelibrary.com/cdsr/doi/10.1002/14651858.CD005950.pub5/full (Accessed February 17, 2026). 10.1002/14651858.CD005950.pub5 PMC7387111 32449959

[58] Scrivener K Dorsch S McCluskey A Schurr K Graham PL Cao Z . Bobath therapy is inferior to task-specific training and not superior to other interventions in improving lower limb activities after stroke: a systematic review. J Physiother. (2020) 66:225–35. doi: 10.1016/j.jphys.2020.09.008 33069609

[59] Lyu D Lyu X Zhang Y Ren Y Yang F Zhou L . Tai chi for stroke rehabilitation: A systematic review and meta-analysis of randomized controlled trials. Front Physiol. (2018) 9:983. doi: 10.3389/fphys.2018.00983 30090071 PMC6068268

[60] Schmalzl L Crane-Godreau MA Payne P. Movement-based embodied contemplative practices: definitions and paradigms. Front Hum Neurosci. (2014) 8:205. doi: 10.3389/fnhum.2014.00205 24782738 PMC3995074

[61] Alt Murphy M Munoz-Novoa M Heremans C Branscheidt M Cabanas-Valdés R Engelter ST . European stroke organisation (ESO) guideline on motor rehabilitation. Eur Stroke J. (2025) 10:1160–88. doi: 10.1177/23969873251338142 40401760 PMC12098312

