# Supplementary material for: Effects of mind-body training on upper-limb function in stroke patients: a multilevel dose-response meta-analysis
Source: Front Med (Lausanne). 2026 Jun 12;13:1827942. doi: 10.3389/fmed.2026.1827942 (PMC13303036; doi:10.3389/fmed.2026.1827942)
Supplement: Supplementary file 2 [file Table_2.docx]

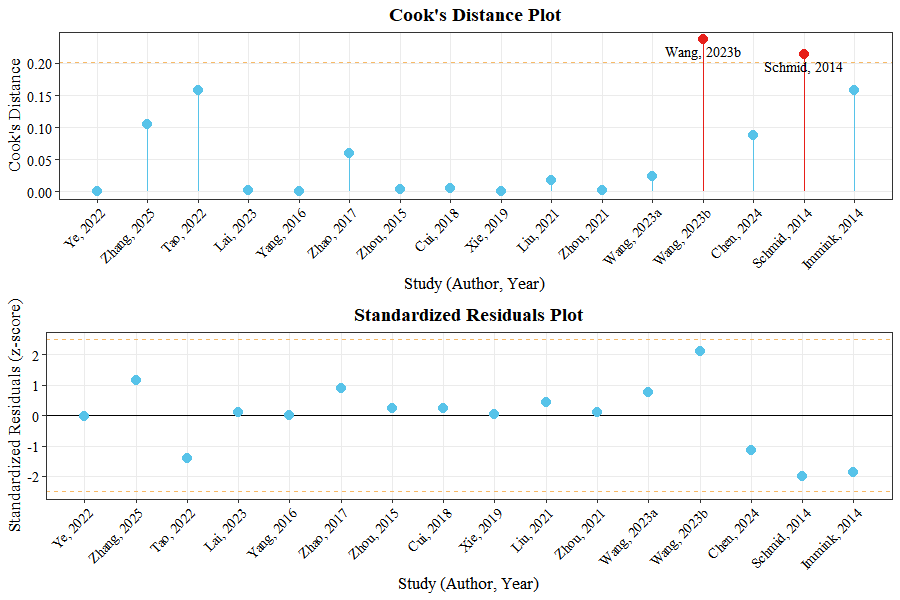


Secondary Influence Analysis of Studies via Standardized Residuals and Cook’s Distance


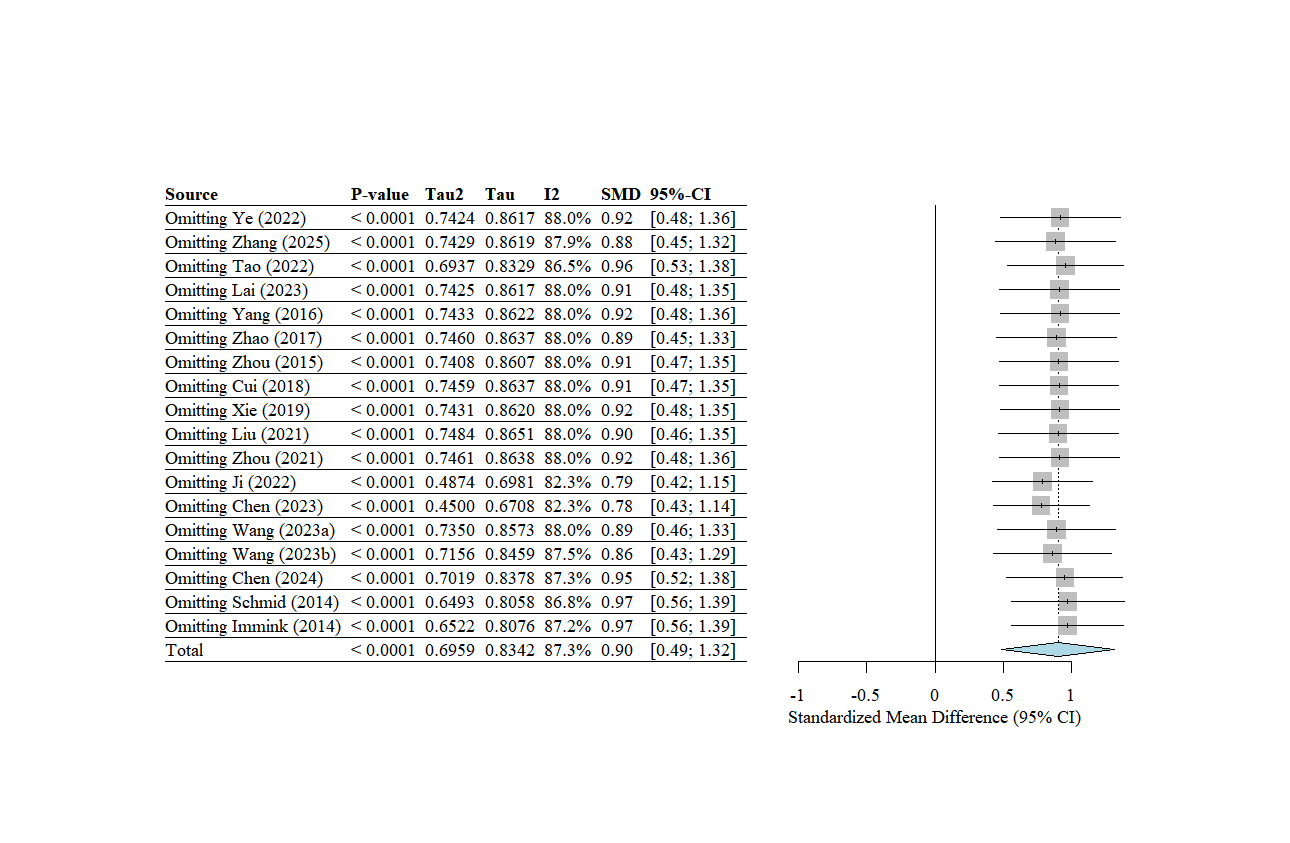


Intervention Endpoint Sensitivity Analysis Plot


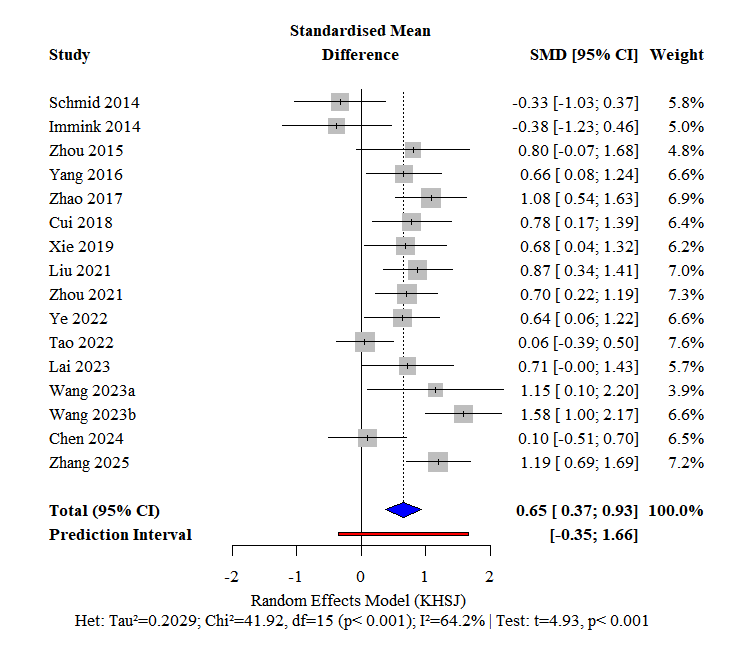


Forest plot of the KHSJ random effects model analysis


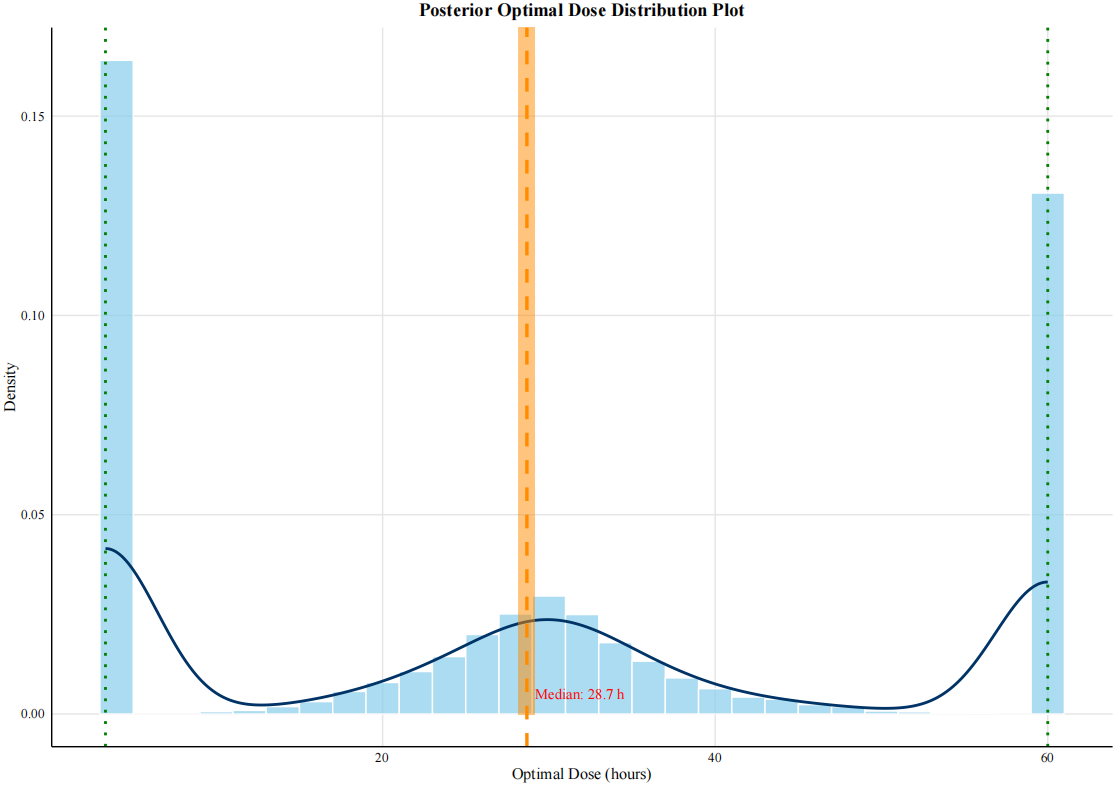

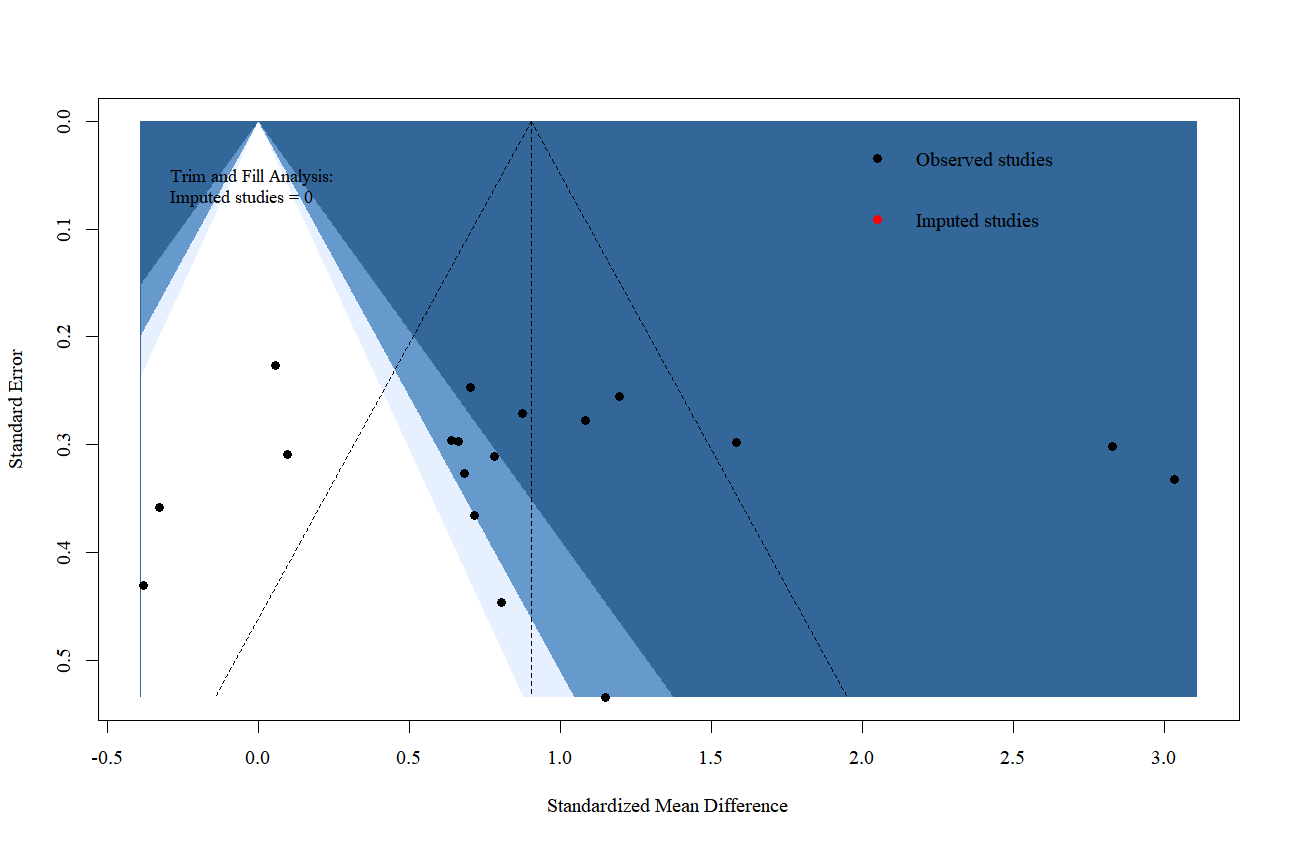
Intervention Endpoint Trim-and-Fill Plot

Posterior Distribution of Optimal Mind-body Training Dose


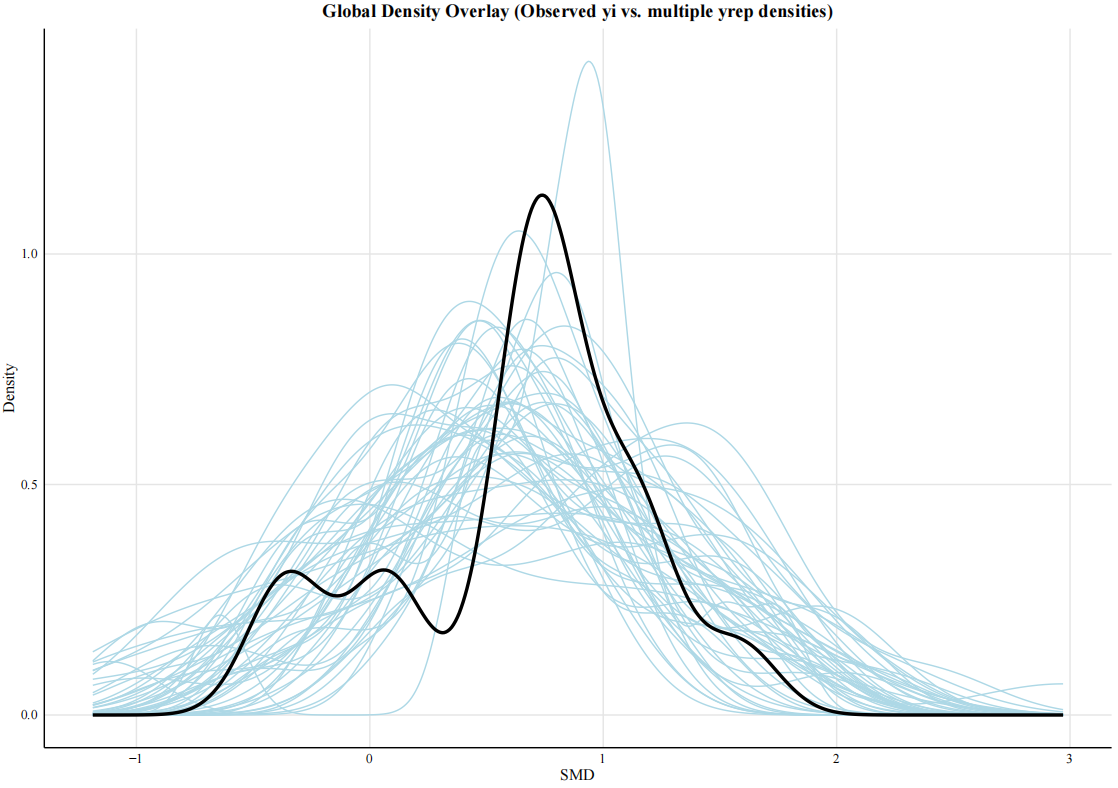


PPC: Density Overlay (Mind-body Training for Upper Limb Function)


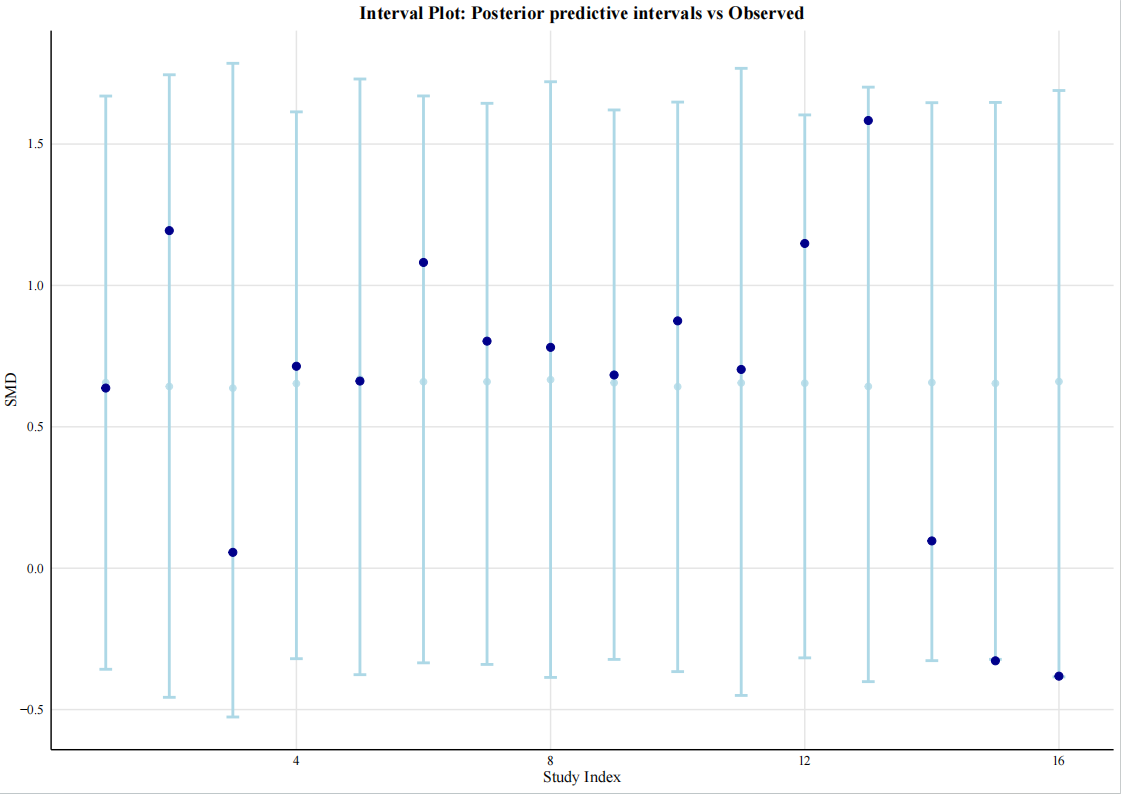
PPC: Observed vs Predicted SMD by Dose


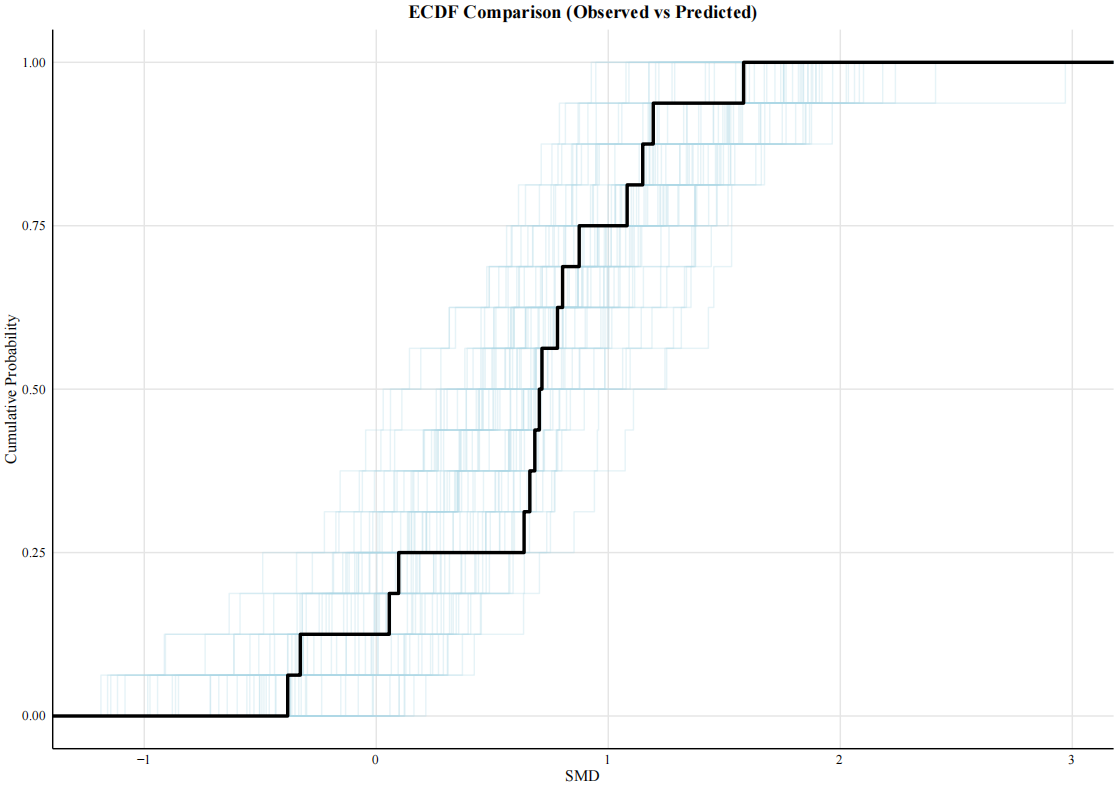


PPC: ECDF Overlay (Mind-body Training for Upper Limb Function)


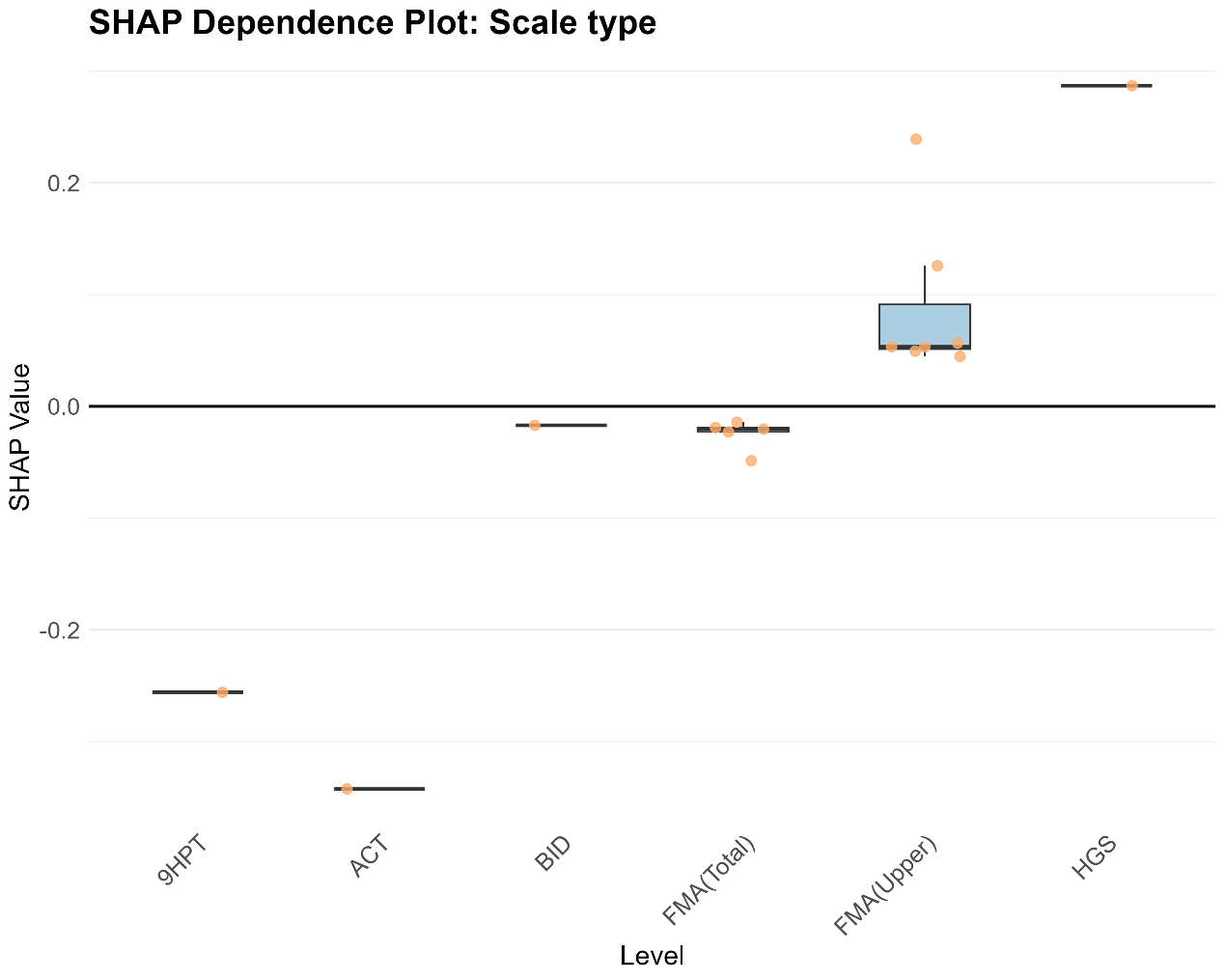
SHAP Dependence Plot:
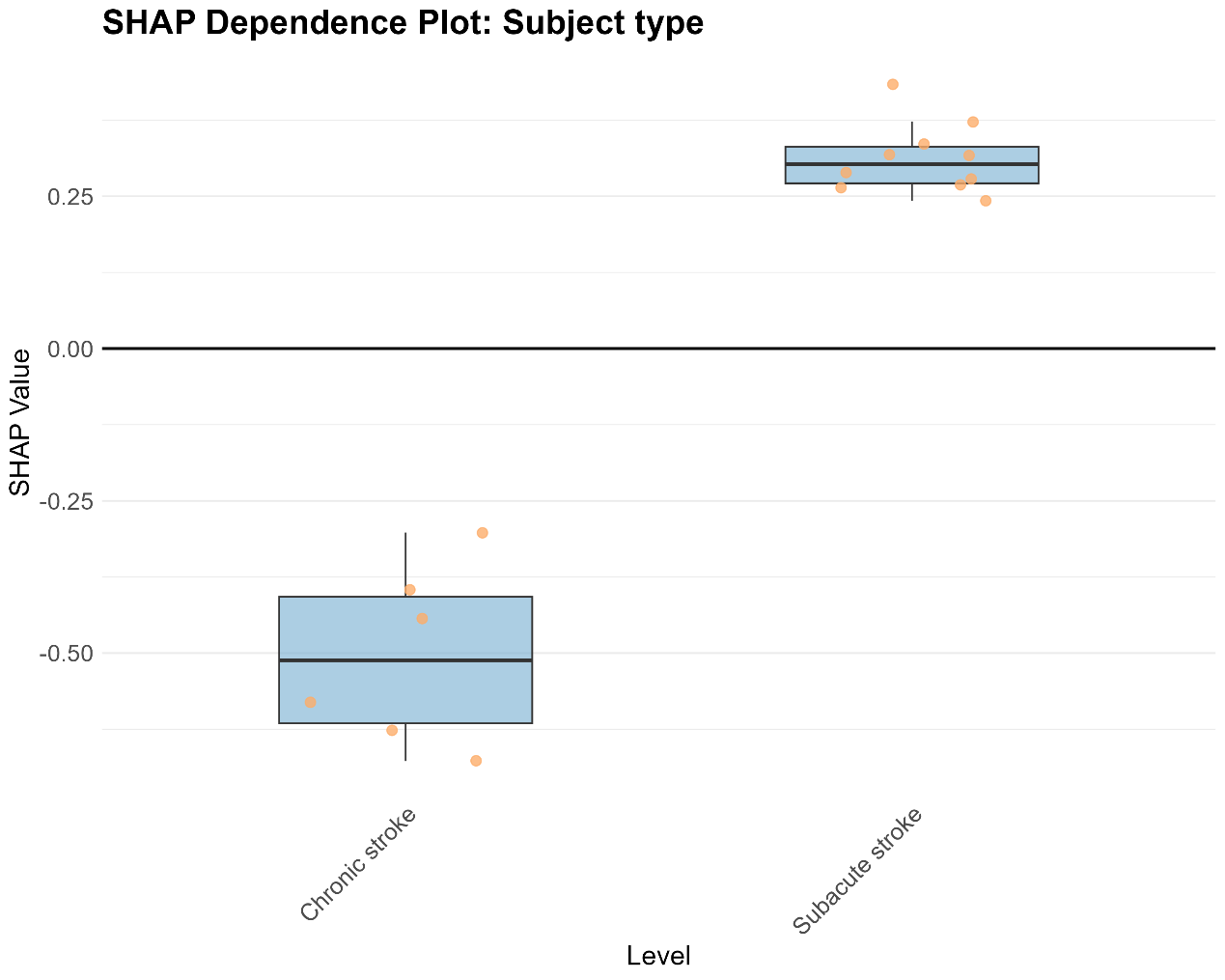
Subject type

SHAP Dependence Plot: Scale type


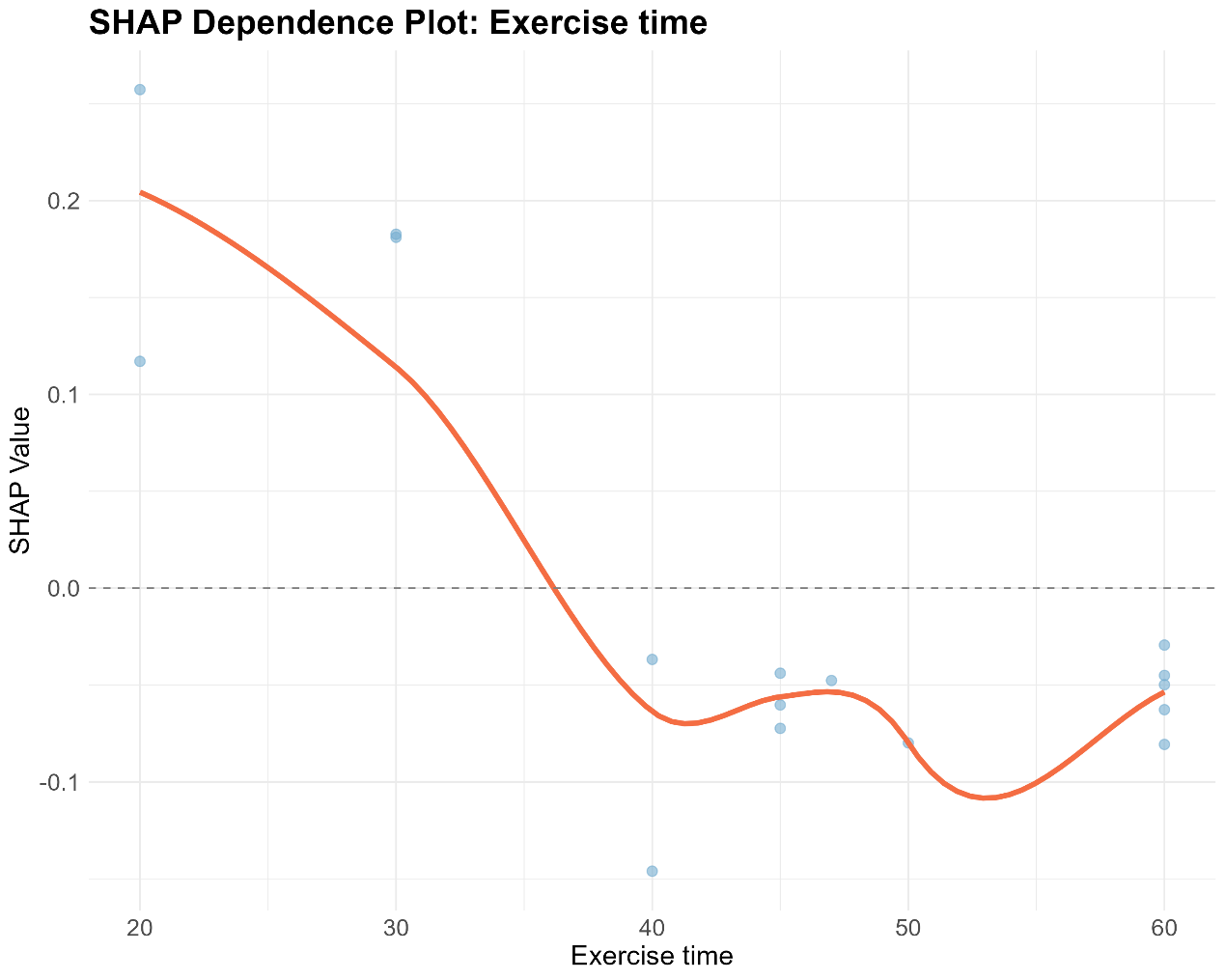


SHAP Dependence Plot: Exercise time


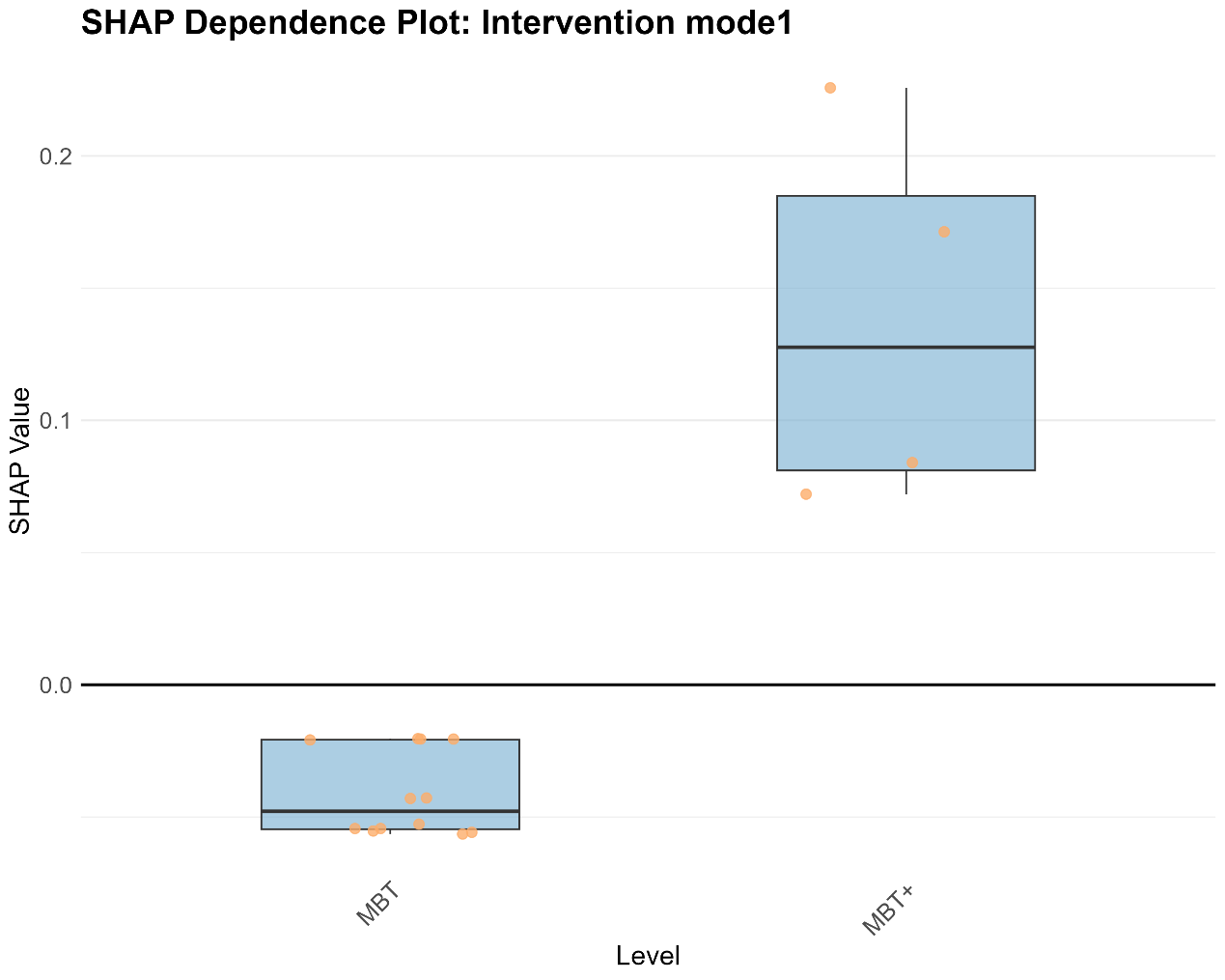


SHAP Dependence Plot: Intervention mode1


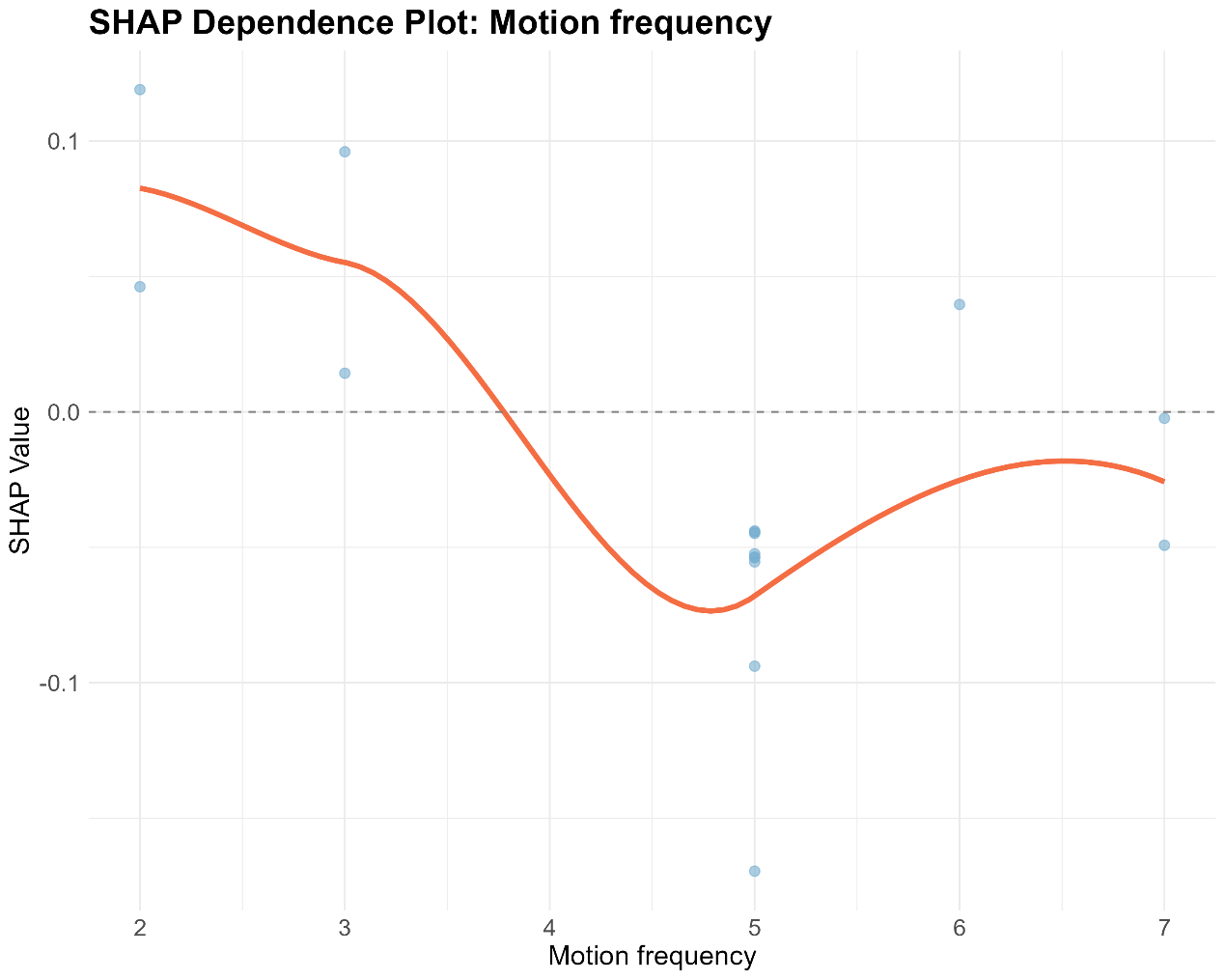


SHAP Dependence Plot: Motion frequency


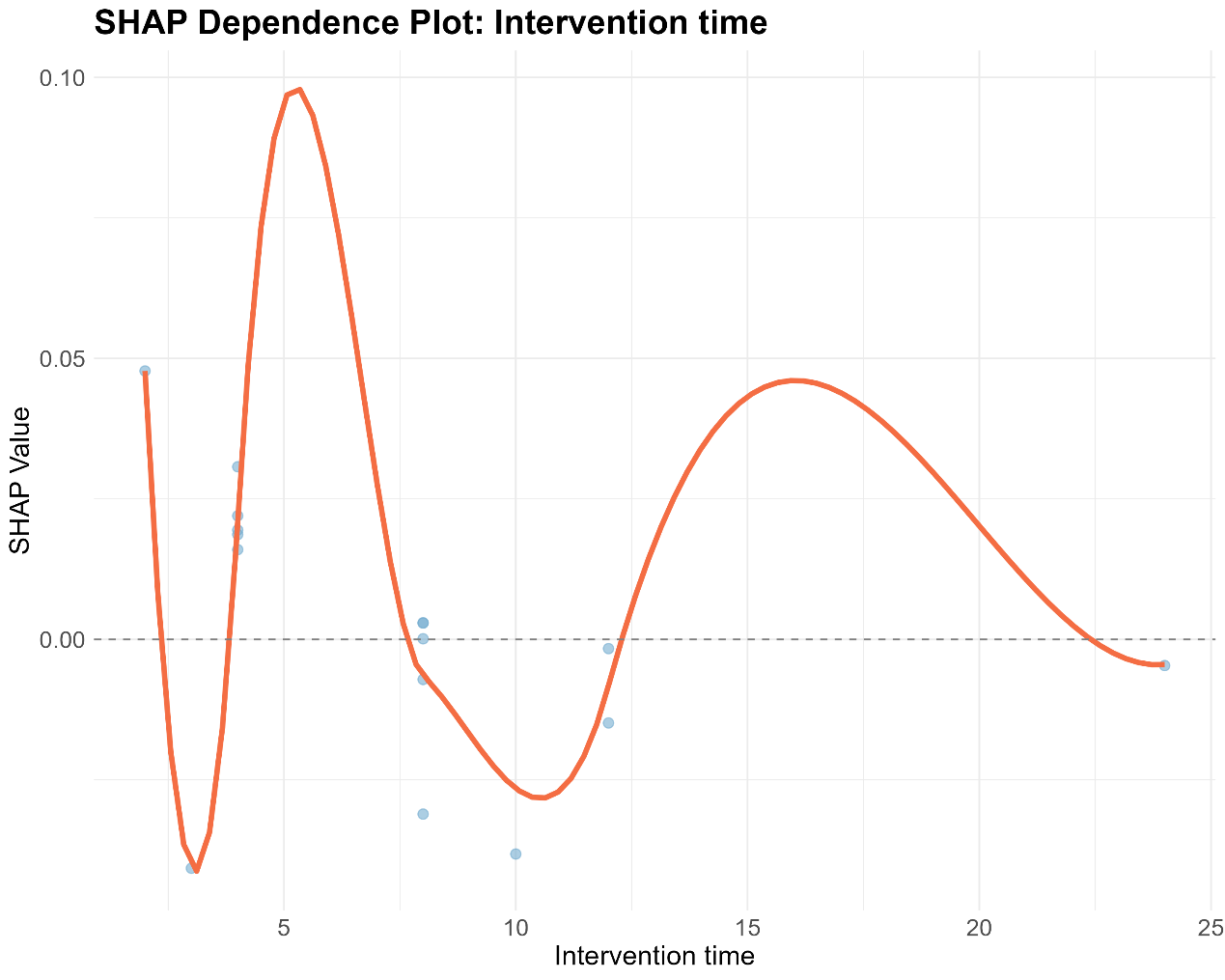


SHAP Dependence Plot: Intervention time


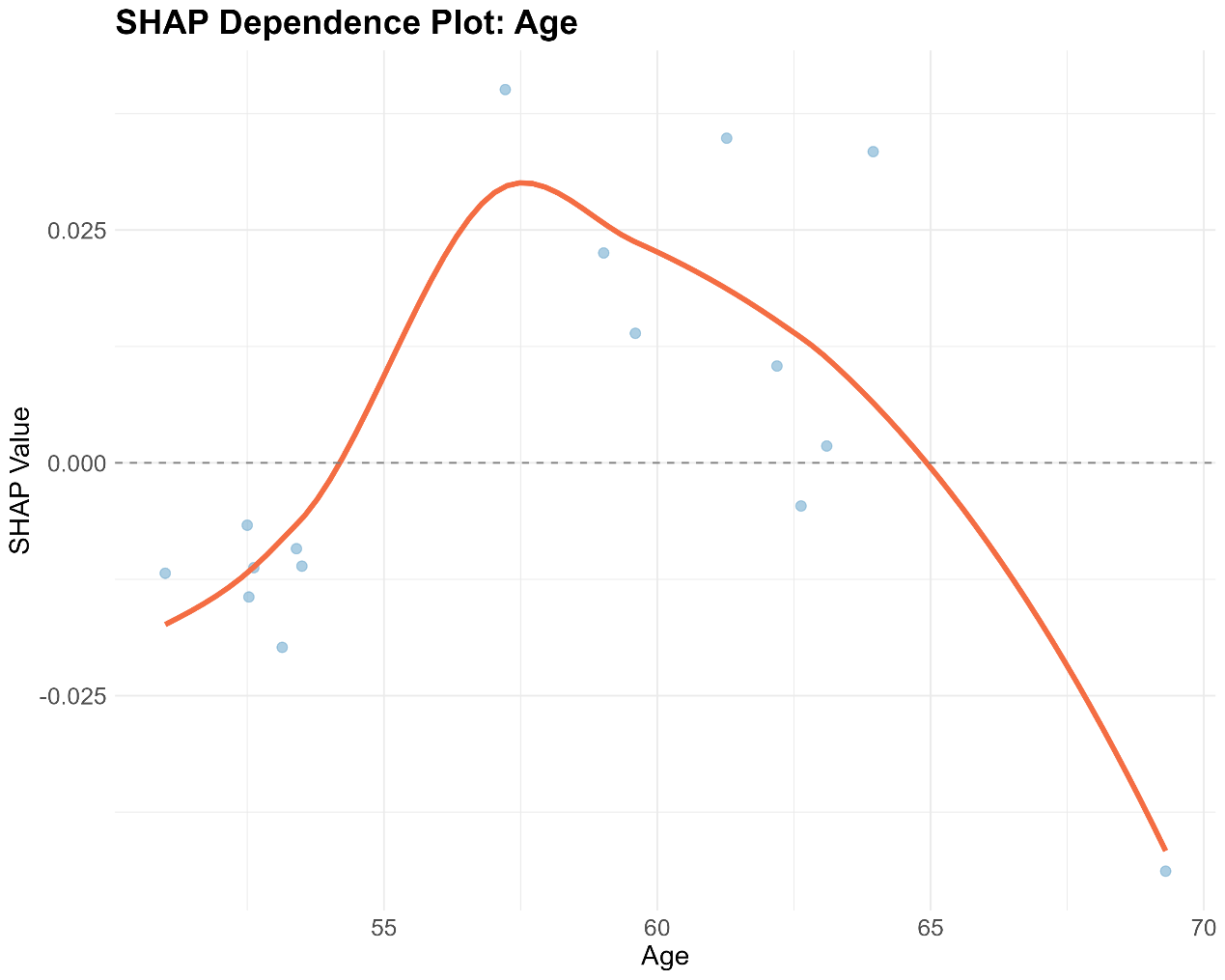


SHAP Dependence Plot: Age


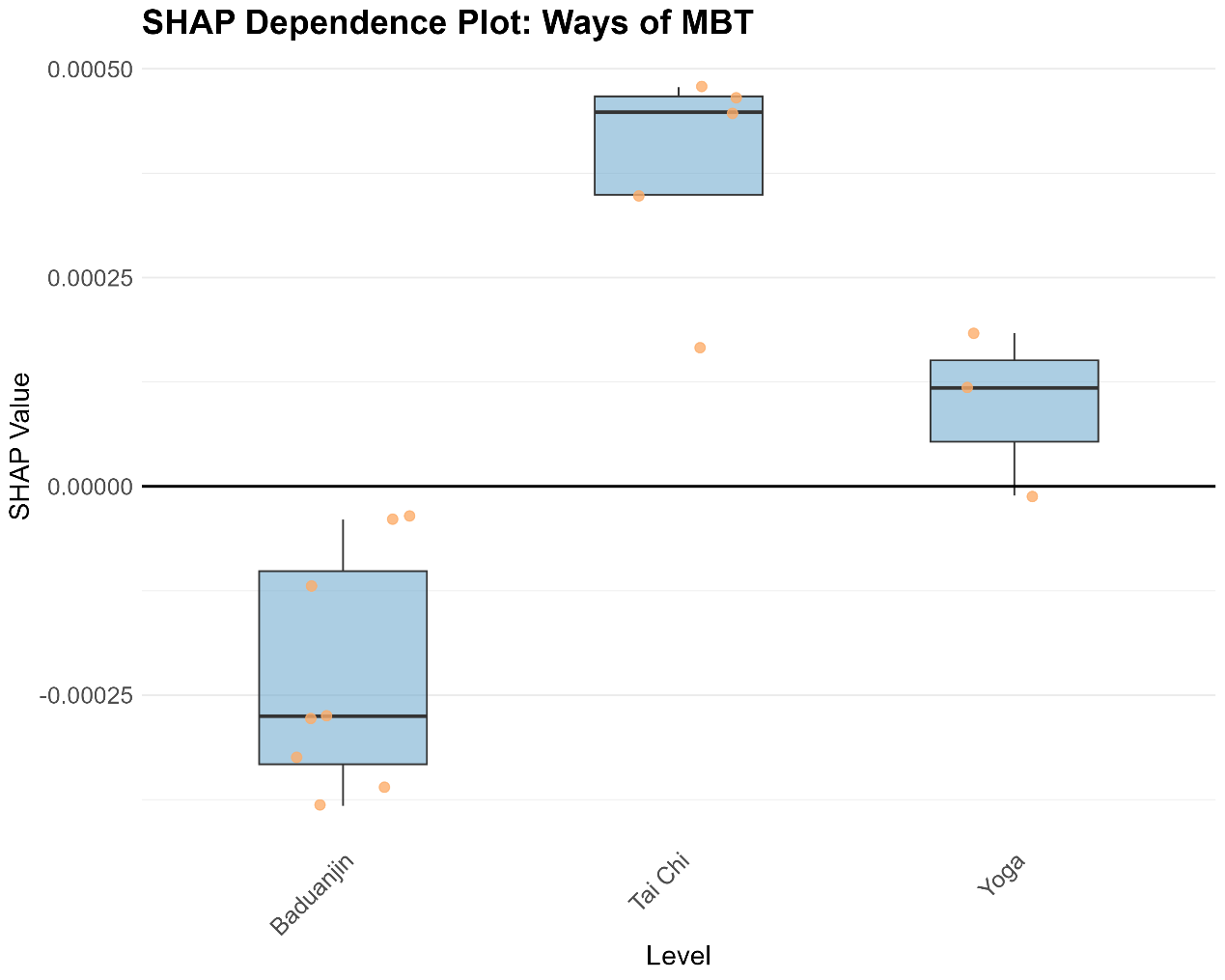


SHAP Dependence Plot: Way of MBT

**Table 2.** Subgroup Analysis Results of Mind-body Training on Upper Limb Function in Stroke Patients

| Dimensionality | sort | K | N | I^2^ | Effect model | SMD and 95%CI | P | P-interaction​​​ |
| --- | --- | --- | --- | --- | --- | --- | --- | --- |
| Intervention frequency/week |  |  |  |  |  |  |  | 0.826 |
|  | < 5 | 4 | 187 | 62.1% | Random | 0.50(-0.01, 1.01) | 0.055 |  |
|  | 5 | 9 | 99 | 54.0% | Random | 0.66(0.39, 0.94) | < 0.001* |  |
|  | > 5 | 3 | 483 | 85.9% | Random | 0.80(-0.39, 1.99) | 0.189 |  |
| Intervention time/week |  |  |  |  |  |  |  | 0.934 |
|  | > 8 | 4 | 319 | 70.2% | Random | 0.61(0.03, 1.18) | 0.039* |  |
|  | < 8 | 7 | 217 | 72.2% | Random | 0.71(0.28, 1.15) | 0.001* |  |
|  | 8 | 5 | 233 | 60.3% | Random | 0.61(0.16, 1.06) | 0.008* |  |
| Exercise time/minute |  |  |  |  |  |  |  | 0.583 |
|  | 40-50 | 7 | 215 | 34.8% | Random | 0.54(0.27, 0.81) | < 0.001* |  |
|  | > 50 | 5 | 248 | 66.8% | Random | 0.64(0.14, 1.14) | 0.012* |  |
|  | < 40 | 4 | 306 | 84.3% | Random | 0.93(0.24, 1.63) | 0.009* |  |
| Subject type |  |  |  |  |  |  |  | < 0.001* |
|  | Chronic stroke | 6 | 269 | 41.6% | Random | 0.16(-0.17, 0.49) | 0.348 |  |
|  | Subacute stroke | 10 | 500 | 0.0% | Random | 0.95(0.75, 1.15) | < 0.001* |  |
| Way of MBT |  |  |  |  |  |  |  | 0.029* |
|  | Baduanjin | 8 | 443 | 66.3% | Random | 0.67(0.33, 1.00) | < 0.001* |  |
|  | Tai Chi | 5 | 225 | 0.0% | Random | 0.99(0.71, 1.28) | < 0.001* |  |
|  | Yoga | 3 | 101 | 63.0% | Random | 0.02(-0.69, 0.73) | 0.965 |  |
| Intervention mode 1 |  |  |  |  |  |  |  | 0.456 |
|  | MBT+ | 4 | 506 | 85.2% | Random | 0.85(0.19, 1.51) | 0.011* |  |
|  | MBT | 12 | 263 | 46.5% | Random | 0.59(0.33, 0.84) | < 0.001* |  |
| Intervention mode 2 |  |  |  |  |  |  |  | 0.111 |
|  | Active Control | 3 | 152 | 60.5% | Random | 1.12(0.49, 1.75) | 0.001* |  |
|  | Usual Care Control | 13 | 644 | 60.9% | Random | 0.56(0.30, 0.83) | 0.001* |  |
| Age |  |  |  |  |  |  |  | 0.969 |
|  | > 60 | 6 | 380 | 82.6% | Random | 0.65(0.10, 1.20) | 0.020* |  |
|  | ≤ 60 | 10 | 389 | 31.8% | Random | 0.66(0.41, 0.91) | < 0.001* |  |
| Scale type |  |  |  |  |  |  |  | 0.800 |
|  | FMA(Upper) | 7 | 308 | 27.9% | Random | 1.20(0.44, 1.04) | < 0.001* |  |
|  | FMA(Total) | 5 | 300 | 59.9% | Random | 0.66(0.30, 1.02) | < 0.001* |  |
|  | Other | 4 | 161 | 86.9% | Random | 0.42(-0.51, 1.36) | 0.376 |  |

**Table 3.** Detailed movement components of the included mind-body training programs

| Study | MBT modality | Intervention dose | Body position/delivery | Specific movements or exercise components reported in the original study | Upper-limb-related components | Trunk / lower-limb / balance components | Breathing/attention / cognitive components | Similarities and differences across studies/reporting completeness |
| --- | --- | --- | --- | --- | --- | --- | --- | --- |
| Ye et al., 2022 | Baduanjin | 24 weeks; 3 days/week; 40 min/session | Group-based community training; 5–10 participants per group; supervised by qualified Baduanjin coaches | Standard Health Qigong Baduanjin based on the 2003 national standard. The program included preparation and ending postures and eight main postures: “Two hands hold up the heavens to regulate the triple energizer,” “Drawing the bow to shoot the hawk,” “Raise one arm to regulate the spleen and stomach,” “Look back to treat five strains and seven impairments,” “Sway the head and shake the tail,” “Two hands hold the feet to strengthen the kidneys and waist,” “Clench fists and glare to increase strength,” and “Rise and fall on tiptoe seven times” | Repeated bilateral arm elevation, bow-drawing movement, unilateral arm lifting, fist clenching, shoulder–elbow–wrist coordination | Weight shifting, trunk flexion/extension, trunk rotation, toe-rising, postural control | Mind-body coordination, breathing regulation, movement awareness, and attention to posture | This study reported the most complete standard Baduanjin sequence and provided a figure of the postures. Similar to other Baduanjin studies, it emphasized slow, coordinated, low-intensity whole-body movement. |
| Zhang et al., 2025 | Body weight-supported Tai Chi Yunshou training | 12 weeks; 5 days/week; 30 min BWS-TCY + 30 min CRT/session | Seated in front of an upper-limb rehabilitation robot; affected forearm fixed to the robotic arm; robot-assisted or resistance/assisted/passive modes | Tai Chi Yunshou movement trajectories were generated and practiced using a rehabilitation robot. The program included customized Yunshou motion paths, visual feedback, soothing music, and different training modes based on impairment severity | Highly upper-limb-focused intervention; repetitive circular Yunshou arm movement; affected upper limb guided by robotic arm; shoulder, elbow, wrist control; hand-eye coordination | Less lower-limb demand because the robotic arm provided weight support; emphasis on stable sitting and controlled upper-limb trajectory | Visual feedback, movement recall, cognitive engagement, hand-eye coordination, attention to movement speed and trajectory | Different from traditional Tai Chi studies, this was a technology-assisted, upper-limb-specific Tai Chi Yunshou program. It reported the training system clearly but not a traditional whole-body Tai Chi sequence. |
| Tao et al., 2022 | Tuina therapy plus qigong guidance | 2 weeks; 5 sessions/week; 20 min/session | Prone position for Tuina; later independent qigong guidance | Tuina involved pressing and kneading acupoints along the Du meridian, including Baihui, Dazhui, Zhiyang, Mingmen, and Changqiang. The therapist rubbed along the line from Changqiang to Dazhui and quickly rubbed along the Du meridian. After treatment, patients were instructed to practice Baduanjin types 1 and 8 | The Tuina component did not include any direct upper-limb exercises. Although the original study referenced the subsequent practice of Baduanjin types 1 and 8, it did not explicitly describe the specific movement sequences and upper-limb-related components. | The description of spine-centered stimulation along the Du meridian has been provided. However, the components related to the trunk, lower limbs, or balance in the subsequent Baduanjin practice were not comprehensively reported. | Manual stimulation, relaxation, qigong-related self-practice, attentional regulation | This study differed from exercise-based MBT studies because the main intervention was manual Tuina therapy rather than active movement training. The Baduanjin component was only briefly described. |
| Lai et al., 2023 | Yoga plus standard rehabilitation | 8 weeks; 2 sessions/week; 60 min/session | Add-on yoga with standard rehabilitation; exact yoga protocol referred to supplementary files | Yoga was described as physical movements combined with breath regulation, postural balance, and meditation. The detailed postures were not fully shown in the main text | Hand grip strength was assessed, but specific hand or upper-limb yoga movements were not fully reported in the main text | Postural balance, standing tolerance, general body movement | Breath regulation and meditation were explicitly described as core yoga components | The article clearly described yoga as a mind-body practice but did not provide sufficient details on the exact yoga postures in the main text. Reporting completeness: partial. |
| Yang et al., 2016 | Tai Chi | 8 weeks; 5 days/week; 40 min/session; 3 supervised and 2 home sessions/week | Standing Tai Chi adapted for hospitalized stroke rehabilitation; video-guided home practice with telephone supervision | Six simplified Tai Chi movements: Qiluoshi, Kaiheshi, Yunshou, Yema Fenzong, Daojuan Gong, and Lanquewei | Repeated bilateral upper-limb movement, arm opening/closing, Yunshou circular arm movement, backward arm movement, grasping/ward-off movement | Step forward, step backward, lateral stepping, center-of-gravity shifting; removal of single-leg standing, deep squatting, pubu, and static fixed postures | Rhythmic, continuous movement; attentional control; slow movement regulation | This was a modified simplified Tai Chi program tailored to Brunnstrom stage and limb activity. It reported named movements and adaptation principles clearly. |
| Zhao et al., 2017 | Tai Chi | 8 weeks; 5 days/week; 30 min/session | Group-based practice in rehabilitation center; guided by martial arts teachers and students; safety protection provided | Six simplified Tai Chi movements: Qiluoshi, Kaiheshi, Yunshou, Yema Fenzong, Daojuan Gong, and Lanquewei. Practice included 5 min relaxation, 10 min six-movement training, 5 min rest, and repeated training | Repetitive arm opening/closing, Yunshou arm coordination, upper-limb and core motor control, coordinated limb movement | Weight shifting, trunk/core control, lower-limb coordination, balance training | Group practice, relaxation, rhythmic movement, psychological engagement | Similar to Yang et al., this used six simplified Tai Chi movements. It was more focused on post-stroke depression and social/psychological participation than on detailed biomechanical description. |
| Zhou et al., 2015 | Modified Tai Chi | 1 month; 5 days/week; 60 min/session | Supervised by one trained intermediate rehabilitation therapist familiar with traditional Tai Chi | Modified international standard Tai Chi movements, including Qishi, Yema Fenzong, Louxi Aobu, Daojuan Gong, Lanquewei, and Yunshou. Patients were not required to follow the full routine sequence but were encouraged to complete the standard posture as much as possible | Bilateral alternating limb movement, arm-reaching, Yunshou, coordination between both sides, hand-eye coordination | Weight shifting, lower-limb support, postural control, balance; attention to eye-follow-hand movement | Relaxation, slow and even movement, coordination of breathing and movement | This study explicitly modified Tai Chi for stroke patients by focusing on postural completion rather than full routine continuity. It reported movement names clearly. |
| Cui et al., 2018 | Health Qigong Baduanjin | 8 weeks; 5 days/week; 45 min/session | 3 supervised sessions and 2 self-practice sessions weekly; coach provided telephone reminders and supervision | Health Qigong Baduanjin. The article described Baduanjin as eight movements, each repeated six times, but the exact movement names were not fully listed in the methods | Arm internal and external rotation throughout the practice; repeated upper-limb movement; coordinated bilateral arm movement | Waist-centered movement, flexible center-of-gravity transfer, whole-body joint and ligament involvement, balance control | Slow, soft, and continuous movement rhythm was described; breathing and attention-related components were not explicitly described in detail. | The exact Baduanjin names were not fully listed, but the article described key movement features, especially waist-axis movement, arm rotation, and repeated practice. |
| Xie et al., 2019 | Baduanjin plus conventional rehabilitation | 3 weeks; 5 days/week; 50 min/session total; Baduanjin after conventional rehabilitation | Therapist-assisted Baduanjin; speed and intensity adjusted according to patient tolerance | Standard eight Baduanjin movements: “Two hands hold up the heavens,” “Drawing the bow,” “Raise one arm to regulate spleen and stomach,” “Look back,” “Sway head and shake tail,” “Two hands hold the feet,” “Clench fists and glare,” and “Rise on toes.” Each movement was practiced three times with 1 min rest between movements | Upper-limb elevation, stretching, unilateral arm lifting, fist clenching, active movement and stretching of upper-limb muscles | Trunk rotation, spine rotation, waist flexion/extension, heel raising, balance and walking-related components | Breathing adjustment, mind-body coordination, therapist-guided intensity control | This study provided detailed Baduanjin movement names and explicit safety adaptations, such as not forcing squatting and allowing reduced upper-limb elevation. |
| Zhou Haiying et al., 2021 | Baduanjin plus rehabilitation training | 3 months; 5 days/week; 60 min/session | Combined with rehabilitation training in elderly convalescent CVA patients | Baduanjin was based on the 2003 Health Qigong Baduanjin standard. The article did not fully list the eight movement names in the methods | The original study identified the intervention as Baduanjin, according to the 2003 Health Qigong Baduanjin standard; however, the specific upper-limb movement components were not explicitly detailed in the original research. | Trunk, lower-limb, balance, and postural-control components were not fully reported for this study. | Breathing, attention, and cognitive components were not explicitly described in detail. | The reporting completeness was limited. Although the intervention was clearly identified as Baduanjin in accordance with national standards, the specific movements and patient-specific modifications were not comprehensively detailed. Furthermore, no additional movement components were inferred beyond those explicitly described in the original study. |
| Liu et al., 2021 | Traditional Baduanjin | 4 weeks; 3 sessions/week; 45 min/session | Observation group practiced Baduanjin; control group did not join any planned fitness activity | Each session included 5 min warm-up, one full set of Baduanjin, 5 min relaxation, a second full set of Baduanjin, and another 5 min relaxation. Exact movement names were not fully listed in the methods | The original study documented the practice of two complete sets of Baduanjin; however, the individual movements and specific upper-limb components were not explicitly detailed within the study. | General Baduanjin practice was reported, but specific trunk, lower-limb, and balance-related components were not individually described. | Warm-up and relaxation were reported; specific breathing, attention, or cognitive components were not fully reported. | This study was primarily focused on post-stroke depression. It reported the session structure clearly but did not fully describe individual Baduanjin movements. |
| Ji et al., 2022 | Baduanjin plus routine rehabilitation | 8 weeks; 6 days/week; 15 min twice daily | Add-on Baduanjin intervention; routine rehabilitation continued | Eight Baduanjin movements were explicitly listed: two hands hold up the heavens; drawing the bow; single arm lifts to regulate spleen and stomach; looking backward; swaying head and tail; two hands hold the feet; clenching fists and glaring; heel rising | Trunk stretching, shoulder/chest muscle training, limb muscle activity, active upper-limb stretch, fist-clenching, arm raising | Cervical movement, waist movement, lumbar/back muscle strengthening, heel movement, whole-body coordination | Breathing, regulation of qi and blood, relaxation, attentional coordination | This article reported detailed movement names and explained the presumed functional role of each movement. It was one of the more detailed Baduanjin reports. |
| Wang Yizhi et al., 2023 | Baduanjin plus action observation therapy | 4 weeks; 6 days/week; 20 min/session | Seated bedside/ward-based training with music, verbal guidance, and video observation | The program used seated upper-limb Baduanjin. It included 5 min preparation, 12 min upper-limb Baduanjin practice, and 3 min finishing relaxation. Patients watched a 12-min action-observation video and imitated the movements; exact Baduanjin movement names were not fully reported | Highly upper-limb-focused seated Baduanjin; affected upper-limb movement, relaxation of hand and fingers, imitation-based movement execution | Mainly seated postural control; limited lower-limb demand | The study reported several interventions, including action observation, imitation, music-guided practice, breathing guidance, and relaxation techniques. However, motor imagery was not explicitly mentioned in the original study. | This study differed from standard Baduanjin studies by combining Baduanjin with action observation therapy. Exact movements were incompletely reported, but delivery procedures were clear. |
| Chen Qing et al., 2023 | Seated Baduanjin plus modern rehabilitation | 3 months; 3–5 sessions/week; 40 min/session | Seated Baduanjin combined with modern rehabilitation training | Seated Baduanjin movements included: sitting upright and relaxed; “embracing Kunlun with both arms”; tapping Yuzhen with fingers; Tianzhu point movement; Jingmen pressing method; left-right turning; supporting/holding the feet; Ren-Du circulation breathing/imagery | Arm circling, bilateral hand movement, finger tapping, upper-limb coordination, wrist/hand involvement | Seated trunk rotation, waist movement, forward bending, foot-reaching movement | Breathing, intention-guided Ren-Du circulation, relaxation, traditional mind-body regulation | This was a seated Baduanjin variant, different from standing Health Qigong Baduanjin. It was adapted for patients with upper-limb dysfunction and reduced balance demands. |
| Wang Wuhao et al., 2023 | Seated Tai Chi | 4 weeks; 7 days/week; 30 min/session | Seated posture; professional demonstration and guidance; heart rate monitored at 40%–60% maximum heart rate | Four seated Tai Chi upper-limb movements: Qiluoshi, Chuanzhangshi, Banlanshi, and Guan’ershi. Each movement was practiced individually for 5 min, followed by 10 min of integrated routine practice | Very upper-limb-specific; vertical, anterior-posterior, lateral, and forward-upward arm movements; shoulder, elbow, wrist, and forearm control; active movement of affected arm with gradual reduction of assistance from the unaffected hand | Seated posture minimized lower-limb influence; emphasis on shoulder flexion, shoulder abduction, elbow flexion, and upper-limb joint dissociation | Breathing guidance, relaxation, attention and intentional control, active participation | This program was specifically designed for Brunnstrom stage II patients. It was more targeted and simpler than traditional Tai Chi routines. |
| Immink et al., 2014 | Yoga | 10 weeks; 1 group class/week, 90 min; home practice 35–45 min on other days | Adapted group yoga plus home practice; practices could be bilateral/unilateral, seated, standing with support, or motor imagery if needed | Yoga asana, pranayama, and Satyananda Yoga Nidra meditation. Group classes included 30 min asana, 10–12 min pranayama, and 20–30 min Yoga Nidra; home practice included 10–20 min asana/pranayama and 25 min Yoga Nidra | Adapted upper-limb postures where appropriate; bilateral or unilateral asana options; motor imagery allowed when physical performance was difficult | Seated or supported standing postures; light-intensity physical activity; sensory and movement awareness; relaxation | Breath awareness, pranayama, meditation, Yoga Nidra, mindful awareness, positive mood | This yoga program reported its structure in detail and allowed substantial individual adaptation. Compared with Tai Chi/Baduanjin, it emphasized meditation and relaxation more strongly. |
| Chen Junwen et al., 2024 | Modified Baduanjin | 4 weeks; 5 days/week; 45 min/session | Therapist-recorded learning video; patients trained after learning movements and breathing; mostly seated with one standing-supported heel-rise movement | Modified Baduanjin included: preparation; two hands hold up the heavens; drawing the bow; single arm lifts to regulate spleen and stomach; looking backward; swaying head and tail; two hands hold the feet; clenching fists and glaring; heel-rising; ending posture. Many actions were performed in sitting and with assistance when needed | Bilateral arm lifting, affected-hand assisted movement, unilateral pushing/pulling, fist-clenching, upper-limb elevation and control | Seated trunk rotation, trunk side-bending, forward flexion, abdominal rubbing, leg/ankle involvement, supported heel-rising | Natural breathing, guided breathing, relaxation, movement awareness | This was a well-described modified Baduanjin program for stroke patients. It preserved the traditional eight-movement structure but adapted posture and assistance for rehabilitation safety. |
| Schmid et al., 2014 | Therapeutic yoga | 8 weeks; 2 sessions/week; 60 min/session | Group therapeutic yoga; standardized and progressive protocol; sitting, standing, floor/mat, and supine positions | Seated practices: slow rhythmic breathing, extended exhalation, eye movements, head/neck movements, scapular ROM, arm movements, finger movements, spinal movements, hip/ankle/foot/toe ROM, alternate nostril breathing. Standing practices: mountain pose, chair pose, locust pose, warrior pose, awkward pose. Floor/supine practices: posterior leg stretches, bridge lifts, knees-to-chest, corpse pose, Yoga Nidra mindfulness meditation | Scapular ROM, arm movements, finger movements, upper-extremity strength-related practice, hand/arm coordination | Standing balance, hip extension, lunges, toe/heel movement, spinal extension/flexion/rotation, lower-limb stretching, endurance-related walking effects | Slow breathing, extended exhalation, alternate nostril breathing, meditation, relaxation, body awareness | This was one of the most detailed yoga protocols. Compared with Baduanjin/Tai Chi, it included more explicit relaxation, meditation, floor/supine postures, and progressive posture categories. |

**Note:** Only the movement components explicitly reported in the original studies were extracted. If the original study did not fully describe the exact movement sequence or exercise components, the corresponding item was marked as 'not fully reported' or 'not explicitly described in the original study.' While some components may be anticipated based on the standard forms of Baduanjin, Tai Chi, or yoga, these components were not coded as reported unless they were explicitly described in the original article.
