# Supplementary material for: Effects of mind-body training on upper-limb function in stroke patients: a multilevel dose-response meta-analysis
Source: Front Med (Lausanne). 2026 Jun 12;13:1827942. doi: 10.3389/fmed.2026.1827942 (PMC13303036; doi:10.3389/fmed.2026.1827942)
Supplement: Supplementary file 3 [file Table_3.docx]

**1. PubMed**

("Stroke"[Mesh] OR "Cerebral Infarction"[Mesh] OR "Cerebral Hemorrhage"[Mesh] OR "Hemiplegia"[Mesh] OR "Cerebrovascular Disorders"[Mesh] OR "Stroke"[tiab] OR "Cerebrovascular Accident"[tiab] OR "CVA"[tiab] OR "Post-stroke"[tiab] OR "Hemiplegia"[tiab] OR "Hemiparesis"[tiab] OR "Brain Ischemia"[tiab] OR "Brain Infarction"[tiab])

AND

("Mind-Body Therapies"[Mesh] OR "Tai Ji"[Mesh] OR "Yoga"[Mesh] OR "Breathing Exercises"[Mesh] OR "Qigong"[Mesh] OR "Mind-body"[tiab] OR "Mind body"[tiab] OR "Tai Chi"[tiab] OR "Taiji"[tiab] OR "Taijiquan"[tiab] OR "Yoga"[tiab] OR "Yogic"[tiab] OR "Baduanjin"[tiab] OR "Ba Duan Jin"[tiab] OR "Eight-section brocade"[tiab] OR "Qigong"[tiab] OR "Chi Kung"[tiab])

AND

("Upper Extremity"[Mesh] OR "upper limb"[tiab] OR "upper-limb"[tiab] OR "upper extremity"[tiab] OR "upper-extremity"[tiab] OR "arm function"[tiab] OR "hand function"[tiab] OR "upper-limb function"[tiab] OR "upper extremity function"[tiab] OR "Fugl-Meyer"[tiab] OR "FMA"[tiab] OR "9-Hole Peg Test"[tiab] OR "9HPT"[tiab] OR "hand grip strength"[tiab] OR "Arm Curl Test"[tiab] OR "Barthel Index"[tiab])

AND

("Randomized Controlled Trial"[pt] OR "Controlled Clinical Trial"[pt] OR "Clinical Trial"[pt] OR random*[tiab] OR "control group"[tiab] OR "RCT"[tiab] OR "placebo"[tiab] OR "randomly"[tiab] OR "trial"[tiab])

NOT

("Review"[pt] OR "Case Reports"[pt] OR "Comment"[pt] OR "Editorial"[pt])

Filters: English or Chinese, Publication date to 2026/01/10

**2. Cochrane Library**

#1 [mh "Stroke"] OR [mh "Cerebrovascular Disorders"] OR [mh "Hemiplegia"] OR "Stroke":ti,ab,kw OR "Cerebrovascular Accident":ti,ab,kw OR "CVA":ti,ab,kw OR "Post-stroke":ti,ab,kw OR "Hemiplegia":ti,ab,kw OR "Hemiparesis":ti,ab,kw

#2 [mh "Mind-Body Therapies"] OR [mh "Tai Ji"] OR [mh "Yoga"] OR [mh "Qigong"] OR "Mind-body":ti,ab,kw OR "Tai Chi":ti,ab,kw OR "Taiji":ti,ab,kw OR "Taijiquan":ti,ab,kw OR "Yoga":ti,ab,kw OR "Baduanjin":ti,ab,kw OR "Ba Duan Jin":ti,ab,kw OR "Eight-section brocade":ti,ab,kw OR "Qigong":ti,ab,kw

#3 [mh "Upper Extremity"] OR "upper limb":ti,ab,kw OR "upper-limb":ti,ab,kw OR "upper extremity":ti,ab,kw OR "upper-extremity":ti,ab,kw OR "arm function":ti,ab,kw OR "hand function":ti,ab,kw OR "upper-limb function":ti,ab,kw OR "upper extremity function":ti,ab,kw OR "motor function":ti,ab,kw OR "Fugl-Meyer":ti,ab,kw OR FMA:ti,ab,kw OR "9-Hole Peg Test":ti,ab,kw OR 9HPT:ti,ab,kw OR "hand grip strength":ti,ab,kw OR "grip strength":ti,ab,kw OR "Arm Curl Test":ti,ab,kw OR ACT:ti,ab,kw OR "Barthel Index":ti,ab,kw

#4 [mh "Randomized Controlled Trials"] OR "randomized controlled trial":ti,ab,kw OR RCT:ti,ab,kw OR "controlled trial":ti,ab,kw OR random*:ti,ab,kw

#5 #1 AND #2 AND #3 AND #4

Publication Date to January 10, 2026, Language: English or Chinese

**3. Web of Science**

Search time range: All years up to January 10, 2026

(TS=("Stroke" OR "Cerebrovascular Accident" OR "CVA" OR "Cerebral Infarction" OR "Cerebral Hemorrhage" OR "Post-stroke" OR "Hemiplegia" OR "Hemiparesis" OR "Brain Ischemia"))

AND

(TS=("Mind-body" OR "Mind body" OR "Tai Chi" OR "Taiji" OR "Taijiquan" OR "Yoga" OR "Yogic" OR "Baduanjin" OR "Ba Duan Jin" OR "Eight-section brocade" OR "Qigong" OR "Chi Kung"))

AND

(TS=("upper limb" OR "upper-limb" OR "upper extremity" OR "upper-extremity" OR "arm function" OR "hand function" OR "upper-limb function" OR "upper extremity function" OR "motor function" OR "Fugl-Meyer" OR FMA OR "9-Hole Peg Test" OR 9HPT OR "hand grip strength" OR "grip strength" OR "Arm Curl Test" OR ACT OR "Barthel Index"))

AND

(TS=("randomized controlled trial" OR RCT OR "controlled trial" OR "clinical trial" OR "random*" OR "placebo"))

NOT

(TS=("review" OR "case report" OR "commentary" OR "editorial" OR "meta-analysis"))

Timespan: All years to 10-January-2026

**4. PsycINFO**

Search time range: All years up to January 10, 2026

1. "cerebrovascular accident" OR stroke OR "brain infarction" OR "brain hemorrhage" OR hemiplegia OR "post-stroke" OR "post stroke" OR hemiparesis

2. "mind body therapy" OR "mind-body therapy" OR "mind-body training" OR "mind-body exercise" OR "tai chi" OR taiji OR taijiquan OR yoga OR yogic OR baduanjin OR "ba duan jin" OR "eight section brocade" OR qigong OR "chi kung" OR meditation OR "breathing exercise"

3. "upper limb" OR "upper-limb" OR "upper extremity" OR "upper-extremity" OR "arm function" OR "hand function" OR "upper-limb function" OR "upper extremity function" OR "motor function" OR "Fugl-Meyer" OR FMA OR "9-Hole Peg Test" OR 9HPT OR "hand grip strength" OR "grip strength" OR "Arm Curl Test" OR ACT OR "Barthel Index"

4. "randomized controlled trial" OR "randomised controlled trial" OR "controlled clinical trial" OR random* OR RCT OR "control group" OR trial

5. 1 AND 2 AND 3 AND 4

Limits: publication date from database inception to January 10, 2026; human studies; journal articles. Language eligibility: English or Chinese full text.
